# Growth hormone significantly increases the adult height of children with idiopathic short stature: comparison of subgroups and benefit

**DOI:** 10.1186/1687-9856-2014-15

**Published:** 2014-07-16

**Authors:** Juan F Sotos, Naomi J Tokar

**Affiliations:** 1 Nationwide Children’s Hospital, The Ohio State University – College of Medicine, 700 Children’s Drive, Columbus, OH 43205, USA; 2 Nationwide Children’s Hospital, 700 Children’s Drive, Columbus, OH 43205, USA

**Keywords:** Idiopathic short stature, Growth hormone, Short children, Short stature

## Abstract

**Background:**

Children with Idiopathic Short Stature do not attain a normal adult height. The improvement of adult height with treatment with recombinant human growth hormone (rhGH), at doses of 0.16 to 0.28 mg/kg/week is modest, usually less that 4 cm, and they remain short as adults. The benefit obtained seems dose dependent and benefits of 7.0 to 8.0 cm have been reported with higher doses of 0.32 to 0.4 mg/kg/week, but the number of studies is limited. The topic has remained controversial.

**Objective:**

The objective was to conduct a retrospective analysis of our experience with 123 children with ISS treated with 0.32 ± 0.03 mg/kg/week of rhGH, with the aim of comparing the different subgroups of non-familial short stature, familial short stature, normal puberty, and delayed puberty and to assess the benefit by comparison with 305 untreated historical controls, from nine different randomized and nonrandomized controlled studies.

**Results:**

Eighty eight of our children (68 males and 20 females) attained an adult height or near adult height of -0.71 SDS (0.74 SD) (95% CI, -0.87 to -0.55) with a benefit over untreated controls of 9.5 cm (7.4 to 11.6 cm) for males and 8.6 cm (6.7 to 10.5 cm) for females.

In the analysis of the subgroups, the adult height and adult height gain of children with non-familial short stature were significantly higher than of familial short stature. No difference was found in the cohorts with normal or delayed puberty in any of the subgroups, except between the non-familial short stature and familial short stature puberty cohorts. This has implications for the interpretation of the benefit of treatment in studies where the number of children with familial short stature in the controls or treated subjects is not known.

The treatment was safe. There were no significant adverse events. The IGF-1 values were essentially within the levels expected for the stages of puberty.

**Conclusion:**

Our experience was quite positive with normalization of the heights and growth of the children during childhood and the attainment of normal adult heights, the main two aims of treatment.

## Introduction

Children with idiopathic short stature (ISS) do not attain a normal adult height. In the three randomized controlled studies (Cochran Central Register Control Trials), the adult height of the controls was -2.4 SDS (0.56 SD) [1], -2.37 SDS (0.46 SD) [2] and -2.2 SDS (0.75 SD) [3]. In an additional 6 non-randomized controlled studies the adult height of the controls ranged between -2.4 SDS (0.8 SD) and -1.88 SDS (0.57 SD) [4-9].

Growth hormone treatment significantly improves the growth velocity and the adult height of children with ISS [10-13] and is considered safe [14-16]. The United States (US) Food and Drug Administration (FDA) approved growth hormone treatment for children with ISS in 2003.

Nevertheless, the use of growth hormone has remained controversial [17-19], mainly because of the modest benefit [20,21] and high cost [22,23]. As Wit JM and Dunkel L stated [24], few topics in pediatric endocrinology provoked more discussion and dissent than ISS. The impression is that despite the significant gain in height, growth hormone treated children remain short as adults, in the lower level of the normal range; an improvement in adult height after years of treatment in the order of 4 cm and a benefit that is less than in other conditions for which GH has been licensed [20,21]. Many of the studies, however, used GH doses of 0.16 to 0.26 mg/kg/week, which may not have been adequate. Furthermore, some of the studies included children with intrauterine growth retardation or with familial short stature, which may have affected the results.

The benefit obtained seems dose dependent and mean benefits of 7.0 to 8.0 cm have been reported with higher doses of 0.32 to 0.4 mg/kg/week [2,3,9].

There have been also ethical issues raised [25] and considerations expressed as to whether ISS should be considered a disease, whether the degree of psychosocial morbidity warrants treatment [26-28], whether it is enhancement or endo-cosmetology rather than treatment [29], and whether treatment has any effect in health related quality of life [30-32].

As the name idiopathic indicates, the cause is unknown. A variety of genes affecting growth, of genes along the growth hormone IGF-I axis [33-38], polygenic traits determined by polymorphisms [39], heterozygous GHR mutations, a dominant negative mutation of the GHR causing familial short stature [40] and mutations in other genes have now been demonstrated in children previously classified as ISS [41-43]. So the label of normal short children may not be appropriate.

All these concerns have been extensively discussed in a number of publications [44] and taken in consideration in the Consensus of the International Pediatric Endocrine Societies [45]. The interest of the child is the primary concern. The main goal of the treatment is the normalization of the height during childhood and improvement of the adult height. Children with a height of less than -2 SDS [45] or a height of more than 2 SDS below their midparental target height, warrant consideration for treatment.

Idiopathic short stature describes a heterogeneous group of children of unknown etiology with variable response to growth hormone [46]. It is defined as short children with a height below -2 SD (2.3 percentile) (and by some authors below the 5^th^ percentile (-1.65 SD)) for age, sex and population group, normal stimulated growth hormone levels and absence of comorbid conditions (systemic disease, bone dysplasias, hormonal deficiencies, dysmorphic syndromes, chromosomal disorders, malnutrition), intrauterine growth retardation or treatment with medications that affect growth (i.e. Ritalin) [45,47].

Specifically, the children with ISS have normal birth weight, and no growth hormone deficiency [45].

The criteria do not include midparental height (MPH). Thus, studies of idiopathic short stature have included two groups of children: those affected with nonfamilial short stature (NFSS) and those with familial short stature (FSS), who may be different in their response to treatment, adult heights, and attainment of MPH. Children with FSS, without treatment, may attain an adult height near or equal to midparental height, but shorter than the normal population [48,49]. It is possible, also, that the modest or small benefit obtained by growth hormone treatment in a number of studies was because many of the children were affected with familial short stature.

After the Consensus on the definition of idiopathic short stature in 1996 [50], ISS also includes what previously was known as constitutional delay of growth and puberty (CDGP). A number of studies have indicated that the adult height attained in children with CDGP, with heights below -2 SDS, is less than the MPH and that they remain somewhat shorter than normal [51,52], some with average heights in the 10^th^ percentile [53] or 5^th^ percentile [54,55].

As the result of the aforementioned, there is consideration of the need to subcategorize the children into different groups: NFSS and FSS and in both, normal puberty and delayed puberty [45,56].

We conducted a retrospective analysis of our experience with 123 children with ISS treated with rhGH, with the aim of comparing the different subgroups and assessing the benefit by comparison with historical controls. As far as we are aware, there are only 2 studies comparing NFSS and FSS [3,9] and no studies of treated children identifying the different subgroups of NFSS, FSS, normal and delayed puberty. The study was approved by the Institutional Review Board of the Hospital and consent and assent approvals were obtained.

### Subjects

Of the 123 children with idiopathic short stature treated with rhGH (0.32 ± 0.03 mg/kg/week – 6 days per week), from the late 80’s to 2012, 88 attained adult or near adult height, 68 males and 20 females. Twenty-seven of the children were lost to follow-up (22 males out of 98, 22%, and 5 females out of 25, 20%). Eight males with NFSS and delayed puberty, who were treated with depo-testosterone with 50 mg a month for 6 months or 100 mg for 3 months to induce pubertal changes, were not included in the results reported, to avoid questions in the interpretation of results, even though it is known that testosterone increases the growth velocity but does not improve adult height [57,58], if any it may decrease the adult height if treatment is prolonged or with higher doses, by advancing bone age (123 children -27-8 = 88).

Of the 68 males, forty-seven were classified as NFSS and 21 (30%) as FSS; 16 females were classified as NFSS and 4 as FSS. The groups were further divided into those with normal puberty and delayed puberty. The duration of treatment was 5.2 ± 2.7 years for the 68 males and 3.5 ± 0.9 for the 20 females, with a range of 2 to 8 years.

All the children met the criteria for ISS with the height below -2.0 SD (except for 1 female who was -1.81), normal stimulated growth hormone levels, normal birth weight and length, and absence of comorbid conditions.

The age at start of treatment ranged between 4.7 years and 16 years, 11.9 ± 3.3 years for the 68 males and 12 ± 1.9 years for the 20 females. The children were seen every 3 months and the rhGH dose was adjusted at each visit. The beginning dose of GH in the late 80’s was 0.22 mg/kg/week 3 times a week, but the dose was increased to 0.3 mg/kg/week or more, because the response was not as good as expected. With the normal weight gain the dose would decrease to 0.28 mg/kg/week and adjustments were made. The dose ranged from 0.28 to 0.4 mg/kg/week with adjustments, for a calculated average dose, throughout the years, of 0.32 ± 0.03 mg/kg/week. The dose was not increased for puberty.

A number of determinations were obtained before the onset of treatment; bone age, CBC, sedimentation rate, chemistry panel, anti-endomysial antibodies, tissue transglutaminase antibodies, glucose, hemoglobin A_1C_, TSH, free T_4_, IGF-I, IGFBP-3 and cortisol; and most of them annually. An MRI of the brain was obtained at the time of the growth hormone stimulation testing on many of them in the past. This is no longer felt needed. Karyotypes were obtained in many. Bone age was determined by Greulich and Pyle standards and adult height predicted by Bayley-Pinneau tables.

Heights were measured by a wall-mounted stadiometer. The heights at the onset of treatment were expressed as SDS for chronologic age. The height SDS was calculated in the usual manner: height of the subject in cm minus the mean height for age or adults divided by the SD in cm from the mean. Adult heights were obtained at 19 ± 2.45 years, range 15.7 to 27.53, for males and at 18.3 ± 2.34 years, range 15 to 24 years, for females. Sixty-seven of the adult heights were obtained by us (76%) and 9 in a doctor’s office (10%). Twelve (14%) were obtained at home, following detailed instructions and were consistent with predicted heights. The last bone age available in our records was 16.0 ± 1.0 years for males and 14.1 ± 0.46 for females, but some adult heights were obtained later. To obtain final adult heights, the potential remaining growth of some children whose heights were obtained before closure of the epiphyses (near adult height) was calculated. These numbers for calculated final adult heights were not included in the numbers reported and it will be addressed later (Additional file 1: Table S1).

Pubertal development was assessed by the method of Tanner. Males with testicular volume of less than 4.0 ml by the age of 14.0 years and females with no breast development by the age of 13 years were classified as delayed onset of puberty. Children whose heights were below the midparental height SDS target range of -1.6 SD were classified as NFSS and those within the target range as FSS. Children whose fathers or mothers were below -2.0 SDS were also classified as FSS. Of the 25 subjects (males and females) with FSS, 9 (36%) had a father or a mother with a height below -2 SD (-2.25 to -2.87 SD). The classification of delayed onset of puberty includes the previously used definition of constitutional delay of growth and puberty (CDGP) in accordance with the international consensus [45].

Target height (MPH) was calculated from the self-reported parental heights by the method of Tanner: (height of the father + height of the mother + 13)/2 for the males and (height of the father + height of the mother – 13)/2 for females and expressed in cm [47]. No addition for secular trend was needed, since there was no or minimal increase in secular trend in the United States National Health Statistics between 1977 and 2000. Midparental height SDS was calculated by the following equation: (father’s height SDS + mother’s height SDS)/√2(1 + r (M,F); r is the correlation between the parent’s height which is 0.3 [50]. The SDS was based on the US National Center Health Statistics of 1977. The values for MPHs SDSs were not different than those calculated by target height, by the method of Tanner, when corrected for assortative mating (correlation between the parent’s heights). The characteristics of NFSS and FSS children treated to adult height are illustrated in Table 1.

**Table 1:** Idiopathic short stature treated with GH, non-familial short stature (NFSS) & familial short stature (FSS)

	**NFSS Mean (SD)**		**FSS Mean (SD)**		**Reference mean (SD)**
**Characteristic**	**Males (#47)**	**Females (#16)**	**Males (#21)**	**Females (#4)**	**Normal**
Age start – yr	11.96 (3.50)	12.08 (1.39)	11.96 (2.81)	12.07 (0.88)	
BA start – yr	9.00 (3.20)	9.39 (1.51)	9.10 (2.91)	10.30 (1.38)	
GH stim peak mean	12.4	10.8	13.1	14.8	>7 ng/ml
range – ng/ml	7.04 to 28.0	7.0 to 19.8	7.1 to 38.5	8.8 to 20.9	>7 ng/ml
GH Freq Samp 12	2.53 (1.08) (#31)		2.49 (1.46) (#11)		M = 2.54 (0.84)
hr mean ng/ml		1.78 (0.64) (#8)		3.02 (#1)	F = 1.96 (0.85)
SMC – U/ml	0.79 (0.43) (#27)	0.87 (0.22) (#6)	1.09 (0.60) (#11)	1.55 (0.04) (#2)	T-I = 1.04 (0.66)
IGF-1 – ng/ml	144 (101) (#19)	166 (63) (#10)	158 (69) (#11)	205 (9) (#2)	T-I = 109–485
					T-II = 174-512
Duration Rx – yr	5.21 (2.69)	3.59 (0.94)	5.30 (2.30)	3.17 (0.88)	

*Abbreviations*: *GH* serum growth hormone, *BA* bone age, *(#)* number, *M* males, *F* females, *T* Tanner stage.

### Lost to follow-up

Twenty-two males (22% of the total of 98) were lost to follow-up: 6 were treated for less than 6 months and 16 were lost at 10 to 14.4 years of age. Five females (20% of the total of 25) were lost to follow-up: 2 were treated for less than 6 months and 3 were lost at 9.4 to 12.5 years of age. All had a good response to rhGH treatment. An analysis of intent to treat to adult height was conducted.

## Methods

Growth hormone from stimulations tests was assayed by monoclonal antibody (Hybritech IRMA or Immulite chemiluminescent) methods that measure selectively the 22 kDa GH and yields values of 64 to 68% of those obtained with polyclonal antibody RIA methods (Additional file 2: Figure S1). All children had values of serum growth hormone of more than 7 ng/ml in one or both stimulation tests or more than 10 ng/ml when a polyclonal antibody RIA method was used (a few were measured by the Kallestad RIA method at the beginning of the study).

Growth hormone secretion was evaluated by 12-hours overnight frequent sampling on a number of the children in the 1990s; we no longer do that.

Plasma levels of IGF-I were obtained by RIA after acid alcohol extraction (Nichol’s Institute Diagnostic, San Juan Capistrano, CA). This method was not available early in the study and a number of the determinations were made as Somadomedin C. Laboratory determinations for CBC, sedimentation rate, glucose, hemoglobin A_1C_, TSH, free T_4_, cortisol and others were performed by standard laboratory procedures.

### Statistics

Paired and unpaired, two-tailed Student *T* test was used to compare the means of the different groups. A p value of 0.05 or less was considered significant. The 95% confidence intervals (CI) of the different results and groups were calculated. Correlations to assess the factors influencing AHs (young age, delayed bone age, distance to MPH, etc.) have been reported by others and were beyond the scope of this presentation.

## Results

**Figure 1:**
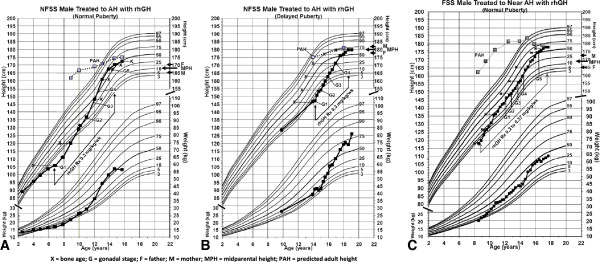
**Growth charts of 3 individual males showing the response to GH treatment.** Growth charts: **A**. Height of a NFSS male with normal puberty treated with rhGH, **B**. Height of a NFSS male with delayed puberty treated with rhGH, and **C**. Height of a FSS male with normal puberty treated with rhGH.

A few growth charts in Figure 1 illustrate what we observed in many children: a catch up growth for the first 3 or more years of treatment to a level expected for the midparental height, a subsequent normal growth, the growth spurt with puberty and the attainment of a normal adult height.

**Figure 2:**
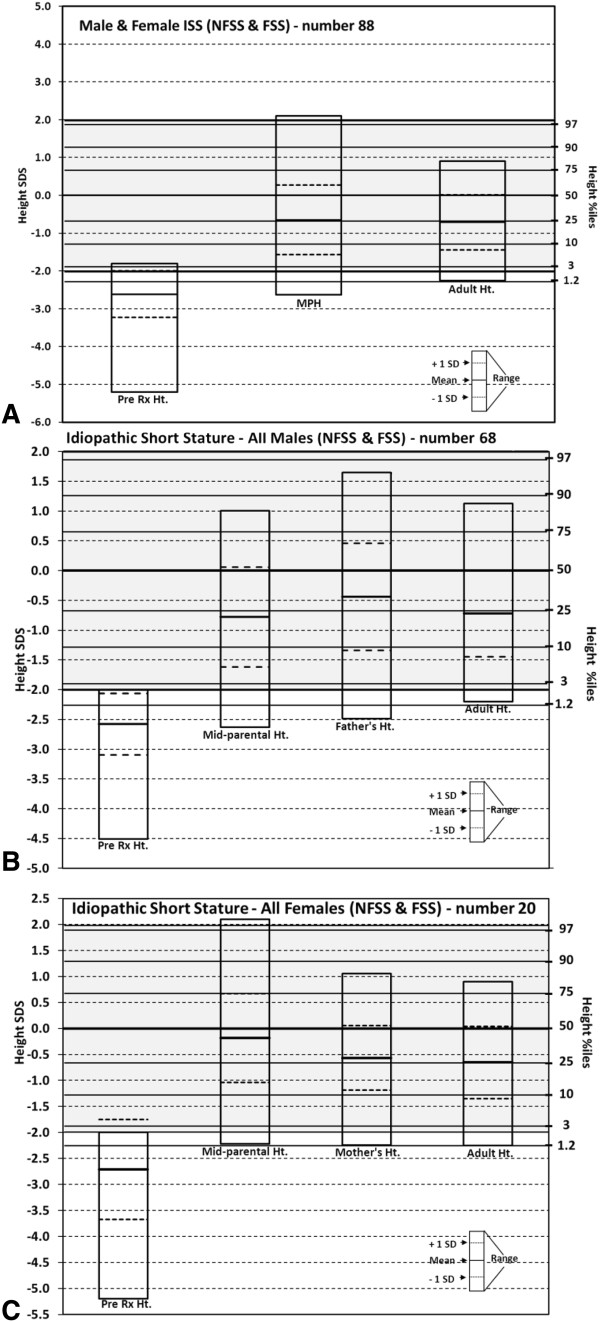
**Height SDSs of all subjects, males and females, before and after treatment.** Height SDS before and after treatment with rhGH: **A**. All ISS males and females, **B**. All ISS males, and **C**. All ISS females. In Table 2 are detailed numbers for means and standard deviations for different measurements to permit comparisons and in Tables 3 and 4 applicable statistics.

Figure 2 provides information on the means ± standard deviations and range of the height at the beginning of treatment, midparental heights, father’s or mother’s heights and adult heights for different groups. (The individual values for the heights at the beginning of treatment, adult heights, midparental heights, age at the beginning of treatment and when the adult heights were obtained, and means ± standard deviations for different subgroups are given in the additional file 3: Figure S2A, B, C, D and Additional file 4: Figure S3A, B).

In Table 2 are detailed numbers for means and standard deviations for different measurements to permit comparisons and in Tables 3 and 4 applicable statistics.

**Table 2:** All ISS (NFSS and FSS) SDS

**Males & Females mean SDS (SD)**	**Treated with GH**			
**Number**	**88**			
Baseline Height (Base H)	-2.61 (0.62)			
MPH	-0.65 (0.92)			
AH (or Near)	-0.71 (0.74)			
AH Gain SDS (AH – Base H)	+1.90 (0.76)			
**Males mean SDS (SD)**	**Total**	**Normal puberty**	**Delayed puberty**	**NP to DP**
**Number**	**68**	**32**	**36**	***p value***
Base H	-2.58 (0.52)	-2.58 (0.63)	-2.58 (0.38)	NS 0.977
MPH	-0.78 (0.84)	-0.94 (0.86)	-0.63 (0.79)	NS 0.141
Fa H.	-0.44 (0.90)	-0.49 (0.96)	-0.39 (0.84)	NS 0.653
AH (or Near)	-0.72 (0.72)	-0.82 (0.77)	-0.64 (0.66)	NS 0.319
AH Gain SDS (AH – Base H)	+1.86 (0.69)	+1.76 (0.67)	+1.94 (0.69)	NS 0.280
**Females mean SDS (SD)**	**Total**	**Normal puberty**	**Delayed puberty**	**NP to DP**
**Number**	**20**	**17**	**3**	***p value***
Base H	-2.71 (0.96)	-2.75 (0.93)	-2.48 (0.44)	NS 0.484
MPH	-0.18 (0.85)	-0.23 (1.08)	+0.09 (0.62)	NS 0.851
Mo H.	-0.57 (0.62)	-0.60 (0.80)	-0.39 (0.94)	NS 0.953
AH (or Near)	-0.65 (0.70)	-0.77 (0.72)	-0.02 (0.82)	NS 0.300
AH Gain SDS (AH – Base H)	+2.06 (0.97)	+1.98 (0.87)	+2.50 (1.23)	NS 0.647
**Males with Non-familial Short Stature (NFSS)**				
**Mean SDS (SD)**	**Total**	**Normal puberty**	**Delayed puberty**	**NP to DP**
**Number**	**47**	**20**	**27**	***p value***
Base H	-2.57 (0.41)	-2.53 (0.47)	-2.60 (0.34)	NS 0.601
MPH	-0.44 (0.73)	-0.58 (0.80)	-0.32 (0.65)	NS 0.262
Fa H	-0.18 (0.76)	-0.22 (0.89)	-0.15 (0.64)	NS 0.758
AH (or Near)	-0.54 (0.64)	-0.60 (0.67)	-0.49 (0.61)	NS 0.603
AH Gain SDS (AH – Base H)	+2.04 (0.64)	+1.94 (0.60)	+2.11 (0.66)	NS 0.372
**Males with Familial Short Stature (FSS)**				
**Number**	**21**	**12**	**9**	***p value***
Base H	-2.59 (0.71)	-2.65 (0.83)	-2.51 (0.49)	NS 0.669
MPH	-1.52 (0.51)	-1.54 (0.57)	-1.49 (0.43)	NS 0.835
Fa H	-0.99 (0.92)	-0.94 (0.90)	-1.06 (0.95)	NS 0.779
AH (or Near)	-1.14 (0.72)	-1.19 (0.79)	-1.07 (0.62)	NS 0.729
AH Gain SDS (AH – Base H)	+1.45 (0.62)	+1.46 (0.68)	+1.44 (0.52)	NS 0.949
**Females with Non-familial Short Stature (NFSS)**				**NP to DP**
**Number**	**16**	**13**	**3**	***p value***
Base H	-2.74 (0.96)	-2.80 (1.04)	-2.48 (0.44)	NS 0.484
MPH	+0.15 (0.85)	+0.16 (0.90)	+0.09 (0.62)	NS 0.851
Mo H	-0.35 (0.62)	-0.34 (0.52)	-0.39 (0.94)	NS 0.953
AH (or Near)	-0.62 (0.70)	-0.77 (0.57)	-0.02 (0.82)	NS 0.300
AH Gain SDS (AH – Base H)	+2.12 (0.97)	+2.03 (0.88)	+2.50 (1.23)	NS 0.647
**Females with Familial Short Stature (FSS)**				
Number	4	4	0	
Base H	-2.59 (0.33)	-2.59 (0.33)		
MPH	-1.49 (0.45)	-1.49 (0.45)		
Mo H	-1.43 (0.97)	-1.43 (0.97)		
AH (or Near)	-0.78 (1.07)	-0.78 (1.07)		
AH Gain SDS (AH – Base H)	1.81 (0.80)	1.81 (0.80)		

**Table 3:** Comparisons SDS (SD) – Probability

		**Mean SDS (SD)**			**Mean SDS (SD)**	***p value***
**All ISS Males & Females (#88)**	AH	-0.71 (0.74)	>	Base H	-2.61 (0.62)	<0.0001
	AH	-0.71 (0.74)	=	MPH	-0.65 (0.92)	NS 0.638
**Males**						
All Males (#68)	AH	-0.72 (0.72)	>	Base H	-2.58 (0.52)	<0.0001
	AH	-0.72 (0.72)	=	MPH	-0.78 (0.84)	NS 0.674
	AH	-0.72 (0.72)	≤	Fa H	-0.44 (0.90)	0.050
Normal puberty (NP) (#32)	AH	-0.82 (0.77)	=	Fa H	-0.49 (0.96)	NS 0.148
Delayed puberty (DP) (#36)	AH	-0.64 (0.66)	=	Fa H	-0.39 (0.84)	NS 0.184
Males NFSS (#47)	AH	-0.54 (0.64)	>	Base H	-2.57 (0.41)	<0.0001
	AH	-0.54 (0.64)	=	MPH	-0.44 (0.73)	NS 0.489
	AH	-0.54 (0.64)	<	Fa H	-0.18 (0.76)	0.019
NP (#20)	AH	-0.60 (0.67)	=	Fa H	-0.22 (0.89)	NS 0.156
DP (#27)	AH	-0.49 (0.61)	≤	Fa H	-0.15 (0.64)	NS 0.057
Males FSS (#21)	AH	-1.14 (0.72)	>	Base H	-2.59 (0.71)	<0.0001
	AH	-1.14 (0.72)	=	MPH	-1.52 (0.51)	NS 0.064
	AH	-1.14 (0.72)	=	Fa H	-0.99 (0.92)	NS 0.587
NP (#12)	AH	-1.19 (0.79)	=	Fa H	-0.99 (0.92)	NS 0.502
	AH	-1.19 (0.79)	=	MPH	-1.52 (0.51)	NS 0.244
DP (#9)	AH	-1.07 (0.62)	=	Fa H	-1.06 (0.95)	NS 0.984
	AH	-1.07 (0.62)	=	MPH	-1.49 (0.43)	NS 0.140
**Females**						
All Females ISS (#20)	AH	-0.65 (0.70)	>	Base H	-2.71 (0.96)	<0.0001
	AH	-0.65 (0.70)	=	MPH	-0.18 (0.85)	NS 0.120
	AH	-0.65 (0.70)	=	Mo H	-0.57 (0.62)	NS 0.728
Females NFSS (#16)	AH	-0.62 (0.70)	<	MPH	+0.15 (0.85)	0.011
	AH	-0.62 (0.70)	=	Mo H	-0.35 (0.62)	NS 0.269
NP (#13)	AH	-0.77 (0.57)	<	MPH	+0.16 (0.90)	0.006
	AH	-0.77 (0.57)	=	Mo H	-0.34 (0.52)	NS 0.067
**Males versus Females**						
All Males vs Females		Males [#68]			Females [#20]	*p value*
	Base H	-2.58 (0.52)	=	Base H	-2.71 (0.96)	NS 0.554
	MPH	-0.78 (0.84)	<	MPH	-0.18 (0.85)	0.025
	Fa H	-0.44 (0.90)	=	Mo H	-0.57 (0.62)	NS 0.568
	AH	-0.72 (0.72)	=	AH	-0.65 (0.70)	NS 0.725
	AH Gain	+1.86 (0.69)	=	Gain	+2.06 (0.97)	NS 0.405
NFSS Males vs Females		Males [#47]			Females [#16]	*p value*
	Base H	-2.57 (0.41)	=	Base H	-2.74 (0.96)	NS 0.485
	MPH	-0.44 (0.73)	<	MPH	+0.15 (0.85)	0.044
	Fa H	-0.18 (0.76)	=	Mo H	-0.35 (0.62)	NS 0.398
	AH	-0.54 (0.64)	=	AH	-0.62 (0.70)	NS 0.239
	AH Gain	+2.04 (0.64)	=	Gain	+2.12 (0.97)	NS 0.969

*Abbreviations*: *AH* adult height, Base *H* baseline height, *MPH* midparental height, *Fa H* father’s height; *Mo H* Mother’s height, *AH Gain* adult height gain, *NS* not significant.

**Table 4:** Comparisons of NFSS versus FSS – SDS (SD) – Probability

		**Mean SDS (SD)**			**Mean SDS (SD)**	***p value***
**Males**		**NFSS (#47)**			**FSS (#21)**	
NFSS vs FSS	Base H	-2.57 (0.41)	=	Base H	-2.59 (0.71)	NS 0.922
	MPH	-0.44 (0.73)	>	MPH	-1.52 (0.51)	<0.0001
	Fa H	-0.18 (0.76)	>	Fa H	-0.99 (0.92)	0.002
	AH	-0.54 (0.64)	>	AH	-1.14 (0.72)	0.003
	AH Gain	+2.04 (0.64)	>	Gain	+1.45 (0.62)	0.001
**Males & Females**		**NFSS (#63)**			**FSS (#25)**	
	Base H	-2.62 (0.60)	=	Base H	-2.59 (0.66)	NS 0.829
	AH	-0.56 (0.65)	>	AH	-1.08 (0.80)	0.008
	AH Gain	2.06 (0.74)	>	Gain	1.51 (0.66)	0.002

All the 88 children with ISS (NFSS and FSS), males and females, attained a mean adult height of -0.71 SDS (0.74 SD) (Table 2), all within the normal percentiles – from the 1^st^ to the 80^th^ percentile for males and for females (Figure 2). All of them attained a height within 2 SD except two children with familial short stature, -2.11 and -2.25 SDS (1.2 percentile). The average height of the 88 children was -0.71 SDS (0.74 SD), significantly different than the baseline height of -2.61 SDS (0.62 SD) (p <0.001) (Table 3), equal to the MPH of -0.65 SDS (0.92 SD) (p, 0.638), and the adult height gain (adult height minus baseline height) was +1.9 SDS (0.76 SD) with a range from +0.29 to +4.13 SDS (95% CI, 1.74 to 2.06).

The 68 males (NFSS & FSS) attained a mean height of -0.72 SDS (0.72 SD), (Table 2) higher than the mean at the onset of treatment of -2.58 SDS (0.52 SD) (p <0.001) (Table 3), equal to the MPH of -0.78 SDS (0.84 SD) (p, 0.674), and equal or less than the father’s height of -0.44 SDS (0.90 SD) (p ≤ 0.05). The adult height gain was 1.86 SDS (0.69 SD), range 0.29 to 3.92 SDS (95% CI, 1.69 to 2.03).

The 20 females attained an adult height of -0.65 SDS (0.70 SD) (Table 2), significantly higher than the baseline height of -2.71 SDS (0.96 SD) (p <0.001) (Table 3), equal to the MPH of -0.18 SDS (0.85 SD) (p, 0.120) and mother’s height of -0.57 SDS (0.62 SD) (p, 0.728). The adult height gain was 2.06 SDS (0.97 SD).

There were no differences between the 68 males and 20 females (Table 3) in regard to baseline height, the SDS for father’s and mother’s heights, adult height of -0.72 SDS (0.72 SD) for males and for females -0.65 SDS (0.70 SD) or for the adult height gain of 1.86 SDS (0.69 SD) versus 2.06 SDS (0.97 SD) (all p values between 0.40 and 0.72). The only difference between the males and the females was in the MPH of -0.78 SDS (0.84 SD) for the males and -0.18 SDS (0.85 SD) for the females (p, 0.025).

The mean adult height of 171.86 ± 4.82 cm was equal to the target height of 172.76 ± 4.34 cm for the males (p, <0.5 > 0.1). Also for the females the mean adult height of 159.68 ± 4.28 cm was equal to the target height of 162.94 ± 4.53 cm (p, <0.5 > 0.1).

### Analysis of the subgroups

#### ***NFSS***

The results obtained are shown in Table 2, applicable statistics in Table 3 and illustrations or graphs in Figure 2. The mean adult height attained by the 47 males was -0.54 SDS (0.64 SD), significantly higher than the baseline height -2.57 SDS (0.41 SD), (p <0.0001), equal to the MPH of -0.44 SDS (0.73 SD) (p, 0.489) and less than the father’s height of -0.18 SDS (0.76 SD) (p, 0.019). The latter statistically different, but it would appear to be of no clinical significance (2.36 cm difference). The adult height of the 20 children with normal puberty, -0.60 SDS (0.67 SD) was equal to the father’s height -0.22 SDS (0.89 SD) (p, 0.156). In the 27 children with delayed puberty the adult height of -0.49 SDS (0.61 SD) was near or equal to the father’s height of -0.15 SDS (0.64 SD) (p, 0.056).

In the 16 female children with NFSS, the adult height was -0.62 SDS (0.70 SD), higher than the baseline height of -2.74 SDS (0.96 SD) (p < 0.001), less than the MPH of +0.15 SDS (0.85 SD) (p, 0.011), and equal to the mother’s height of -0.35 SDS (0.62 SD) (p, 0.269).

The adult height gain for the 47 males with NFSS was 2.04 SDS (0.64 SD), range 0.94 to 3.92 SDS, equal to the adult height gain for the 16 females, 2.12 SDS (0.97 SD) (range 0.99 to 4.13 SDS) (p, 0.969).

#### ***FSS***

The results obtained in the 21 males and 4 females with FSS are shown in Table 2, applicable statistics in Table 3 and illustrations in Figure 2.

The adult height attained by the 21 males was -1.14 SDS (0.72 SD), significantly higher than the baseline height of -2.59 SDS (0.71 SD) (p <0.001), equal to the MPH of -1.52 SDS (0.51 SD) (p, 0.64), equal to the father’s height of -0.99 SDS (0.92 SD) (p, 0.587), and the adult height gain was +1.45 SDS (0.62 SD), range +0.29 to 2.40 SDS (95% CI, 1.17 to 1.73).

The number of females with FSS was only 4 and the values may not accurately represent the values of a larger group. The adult height was -0.78 SDS (1.07 SD), higher than the baseline height of -2.59 SDS, equal or higher than the MPH -1.49 SDS, and equal or higher than mother’s height of -1.43 SDS (0.97 SD). The adult height gain was 1.81 SDS (0.80 SD), range 0.66 to 2.82 SDS.

#### ***Comparison between NFSS and FSS***

The values are in Table 2, statistics in Table 4 and illustrations in Figure 2.

For the 47 males with NFSS and 21 with FSS, there was no difference in the baseline height of -2.57 SDS (0.41 SD) versus -2.59 SDS (0.71 SD) (p, 0.922). The adult height of -0.54 SDS (0.64 SD), 173.1 cm, of the NFSS males was higher than the adult height of males in FSS of -1.14 SDS (0.72 SD) 169.1 cm (p, 0.003). Also the MPH, the father’s height and the gain in height were higher in the NFSS than in the FSS, Table 4, with all the p values of less than 0.002. The adult height gain for the 47 NFSS was 2.04 SDS (0.64 SD), range 0.94 to 3.92 SDS, higher that the 1.45 SDS (0.62 SD), range 0.29 to 2.40 SDS, for the 21 FSS children (p, 0.001).

Similar results were obtained when the males and females were grouped, Table 4. There was no difference in the baseline heights, -2.62 SDS (0.60 SD) and -2.59 SDS (0.66 AD), (p, >0.5), for the 63 males and females with NFSS and 25 with FSS. The adult height of -0.56 SDS (0.65 SD) and adult height gain of +2.06 SDS (0.74 SD) for the 63, 47 males and 16 females, with NFSS were higher than the adult height of -1.08 SDS (0.8 SD) and adult height gain of 1.51 SDS (0.66 SD) for the 25, 21 males and 4 females, with FSS (p < 0.01 and <0.001, respectively).

The adult height gain indicates the response to treatment and ranged from 0.29 to 3.92 SDS. The variability in the response to treatment with GH in ISS is well known. This variability was seen in NFSS and FSS. Of interest, however, was that the response to treatment was less in FSS (0.29 to 2.4 SDS) than in NFSS (0.94 to 3.9 SDS).

For the females the number with FSS is small, 4, the result are probably not an accurate reflection of the group and statistics could be doubtful (false results). Nevertheless, comparisons with the 16 females with NFSS were made. The baseline height for the NFSS was equal to the FSS (p, 0.556). The MPH was higher in the NFSS +0.15 SDS (0.85 SD) than in the FSS, -1.49 SDS (0.45) (p, 0.001). There was no difference in the mother’s heights, adult heights (159.9 cm vs 158.9 cm) or adult height gain (Table 4, all p values from 0.147 to 0.992).

### Variability of the Response to Treatment with Growth Hormone

We definitely observed the variability in response. The AH gain of all the 88 children (males and females) was +1.9 SDS (0.76 SD) with a range for +0.29 to +4.13 SDS, quite a range, (95% CI 1.71 to 2.06). AH was -0.71 SDS (0.74 SD), that would give a range, ± 2 SD, of -2.19 SD to -0.77 SD (page 12). Range of Ahs could be seen in Additional file 3: Figure S2a, b, c, and Additional file 4: Figure S3a and b.

For the 68 males (non-familial and familial short stature), the AH gain was 1.86 SDS (0.69 SD) with a range of +0.29 to 3.92 SDS (95% CI, 1.69 to 2.03). The AH was -0.72 SDS (0.72 SD) giving a range of ± 2 SD of -2.16 to +0.72 SDS.

The AH gain for the 47 males with NFSS was 2.04 SDS (0.64 SD) with a range of 0.94 to 3.92 SDS. The AH gain for the 21 males with FSS was 1.45 SDS (0.62 SD) with a range of 0.29 to 2.40 SDS (p = 0.001).

The AH was -0.54 SDS (0.64 SD), for a range ± 2 SD of -1.82 to +0.74 SDS for the 47 males with NFSS. For the 21 males with FSS the AH was -1.14 SDS (0.72 SD) for a range of ± 2 SD of -2.58 to +0.3 SDS (p = 0.003).

### The effect of puberty

The comparison of the values for different measures obtained for all the children with normal puberty and delayed puberty, males 68 and 20 females, in all the subgroups, NFSS and FSS, males and females are shown in Table 2. There was no difference in any of the values for the baseline height, MPH, father’s height, mother’s height, adult height, and gain in height, for any of the subgroups, for normal and delayed puberty cohorts; all the p values were more than 0.05. There was a difference, however, between NFSS and FSS for normal and delayed puberty.

### The effect of age at start of treatment and duration of treatment

In the study of Rekers-Mombarg, et al. [48] comprising 132 children with ISS, the children declined gradually in growth from a length of -0.8 SDS in boys and -1.3 SDS in girls at birth, to a height of -2.7 SDS at 16 years in boys and 13 years in girls, and increasing to a mean of -1.5 SDS in boys and -1.6 SDS in girls. The gain in SDS from childhood height to adult height (adult height gain) varied from 0.1 SDS (1.22 SD) at 3 years to 0.1 SDS (0.60 SD) at 14 years, and to 1.2 for boys at 16 years and 1.1 for girls at 13 years.

It became of interest to know if the changes that we observed in adult height and adult height gain of our treated subjects were somewhat related to age and not to treatment, even though we did not see differences in our children with normal puberty (usually younger) and those with delayed puberty (usually older). The effect of age on adult height and adult height gain in males with NFSS and FSS (for whom we had adequate numbers) is shown in Table 5. There was a significant difference in the age of children with NFSS with normal puberty (8.6 (2.7 SD) years) and delayed puberty (14.4 (1.0 SD) years) (p < 0.001), but there was no difference in the adult height or adult height gain (p >0.6 and >0.3, respectively).

**Table 5:** Effect of Age on Treated Children with ISS

**Mean (SD) [min to max]**	**Number**	**Treatment start age (yrs)**	**Stop age (yrs)**	**Baseline height SDS (SD)**	**Adult height SDS (SD)**	**Adult height gain SDS (SD)**
**Males – NFSS**						
Normal puberty	20	8.6 (2.76) [5.0 to 13.4]	16.4 (0.97) [14.7 to 18.3]	-2.53 (0.47) [-3.76 to -1.98]	-0.60 (0.67) [-1.73 to 0.81]	1.94 (0.60) [0.94 to 3.01]
Delayed puberty	27	14.4 (1.10) [12.4 to 16.3]	17.9 (0.84) [16.0 to 19.4]	-2.60 (0.34) [-3.37 to -2.00]	-0.49 (0.61) [-1.36 to 1.13]	2.11 (0.66) [1.30 to 3.91]
		p <0.001		p, 0.601	p, 0.603	p, 0.372
**Males – FSS**						
Normal puberty	12	10.3 (2.47) [4.7 to 13.7]	16.9 (0.77) [15.7 to 18.1]	-2.65 (0.83) [-4.51 to -1.98]	-1.19 (0.79) [-2.20 to 0.22]	1.46 (0.68) [0.29 to 2.40]
Delayed puberty	9	14.1 (1.31) [11.6 to 15.9]	17.6 (0.65) [16.9 to 18.7]	-2.51 (0.49) [-3.26 to -1.96]	-1.07 (0.62) [-2.20 to 0.24]	1.44 (0.52) [0.86 to 2.20]
		p <0.001		p, 0.669	p, 0.729	p, 0.949
**Females – NFSS**						
Normal puberty	13	11.4 (1.21) [9.6 to 13.3]	15.5 (0.81) [13.5 to 16.7]	-2.80 (1.04) [-5.20 to -1.81]	-0.77 (0.57) [-1.84 to 0.09]	2.03 (0.88) [1.04 to 4.13]

*Abbreviations*: *min* minimum, *max* maximum.

Similarly, there was a significant difference in the age of children with FSS with normal puberty (10.3 (2.47 SD) years) and delayed puberty (14.1 (1.31 SD) years) (p < 0.001), but no difference in the adult height and adult height gain (p, 0.729 and 0.949, respectively).

In the 13 young females with NFSS, the adult height and adult height gain was the same as in young males with NFSS.

We correlated AH gain and age at the start of treatment and AH gain and duration of treatment in our 47 NFSS males and 21 FSS males, and there was no significant correlation (correlations shown in supplemental graphs in Additional file 5: Figure S4.

The gain in height is very variable for individuals and averages are not accurate or useful to predict the benefit that an individual will obtain. Based on the averages, Table 5, a male with delayed puberty and NFSS with treatment for 3.5 years could gain 2.11 SDS (16 cm in the USA). The gain in height depends on the pubertal growth, benefit of growth hormone, and bone age. In the aforementioned case, Table 5, depending on the age, bone age, progress of bone age, pubertal growth, and growth hormone response, the gain in height could range from 1.3 SDS to 3.9 SDS (10 to 29 cm). The only way that we could inform the subjects of the benefit that he or she could obtain, is the way that we do it now. Based on the height and the bone age, the predicted adult height is obtained. The subject can be informed that based on our experience, growth hormone should be of benefit to him or her, to improve the adult height. The benefit could be 5 cm, 7.5, 10 cm or more but cannot be predicted exactly, because it depends on the response to treatment, progress of bone age, and years of treatment. Then the provider and the subject decide whether treatment is continued to attain the maximum height or ended when he or she is satisfied with the height.

### Analysis of intent to treat to adult height

Children were observed for a period of time prior to treatment, so that their growth rates could be assessed. In a retrospective review we could know the children who were lost to follow up and, consequently, analyze their growth rates prior to and during treatment.

**Figure 3:**
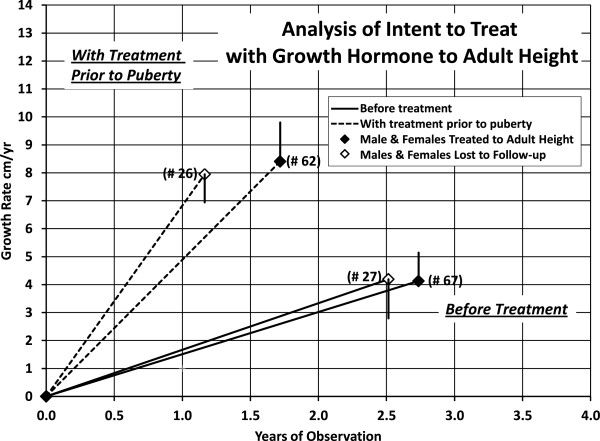
(Analysis of intent to treat).

Growth rates determined for less than 6 months were not included. The growth rates in centimeters per year and the change in the height SDS, prior to treatment, on treatment prior to puberty, and on treatment during the first two years of puberty were not different for treated children lost to follow-up and for those treated to adult height, (p >0.05) (Figure 3 and Table 6). Thus, there was no bias on the reported effect of GH on children treated to adult height.

**Table 6:** Analysis of Intent to Treat to Adult Height Treatment Response with rhGH

	**Number**	**Years observation (Mean SDS ± SD)**	**Growth rate cm/yr (Mean SDS ± SD)**	**Δ Height SDS (Mean SDS ± SD)**
**Males pretreatment**				
Lost to follow-up	22	2.46 ± 1.87	4.29 ± 1.42	-0.31 ± 0.48
Treated to AH	50	2.42 ± 1.37	4.00 ± 1.02	-0.33 ± 0.54
			p, 0.426	p, 0.902
**On treatment with rhGH**				
**Before puberty**				
Lost to follow - up	21	1.20 ± 1.02	7.92 ± 0.94	0.53 ± 0.41
Treated to AH	41	1.83 ± 1.51	8.31 ± 1.44	0.71 ± 0.57
			p, 0.207	p, 0.179
**During puberty**				
Lost to follow - up	7	1.34 ± 0.75	9.09 ± 2.24	0.50 ± 0.28
Treated to AH	50	2.05 ± 1.04	8.31 ± 1.74	0.72 ± 0.48
			p, 0.404	p, 0.116
**Females pretreatment**				
Lost to follow-up	5	2.76 ± 1.54	3.78 ± 1.40	-0.56 ± 0.49
Treated to AH	17	3.71 ± 2.34	4.56 ± 0.71	-0.50 ± 0.37
			p, 0.293	p, 0.810
**On treatment with rhGH**				
**Before puberty**				
Lost to follow - up	5	1.01 ± 0.25	8.06 ± 1.36	0.48 ± 0.32
Treated to AH	10	1.20 ± 0.51	8.94 ± 0.92	0.58 ± 0.27
			p, 0.241	p, 0.593
**During puberty**				
Lost to follow - up	2	1.02 ± 0.03	9.34 ± 2.07	0.72 ± 0.58
Treated to AH	20	1.94 ± 0.97	7.30 ± 1.77	1.05 ± 0.45
			p, 0.382	p, 0.559

### Safety and IGF-1 levels

Complaints of myalgia or arthralgia of the legs early in the treatment, which promptly subsided without adjustment of the GH dose, were very rare. Two of the 123 children had a low serum TSH and free T_4_ (subclinical hypothyroidism) and were treated with levothyroxine, which was discontinued after cessation of treatment.

There were no other side effects.

**Figure 4:**
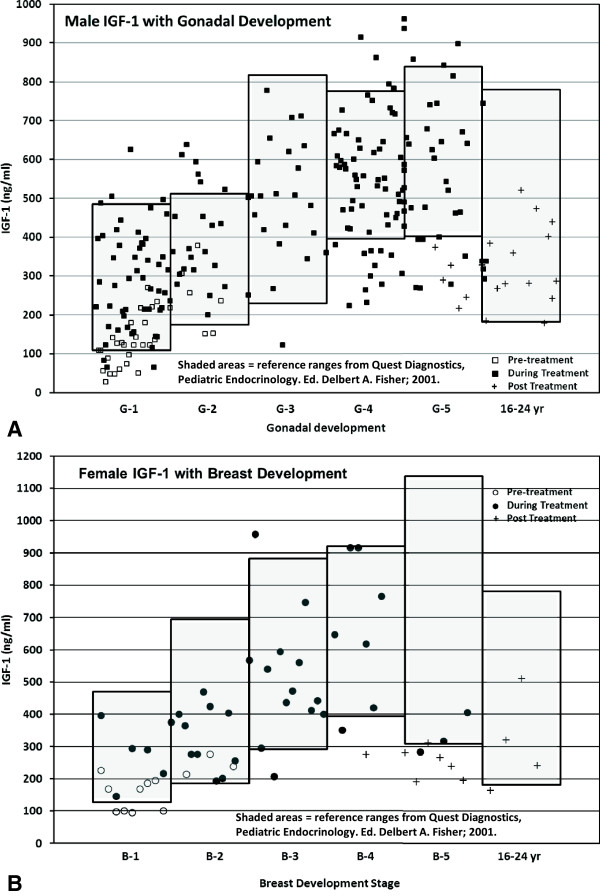
**IGF-1 levels pretreatment, during GH treatment & post treatment.** Serum levels of IGF-1 before, during and after treatment with rhGH: **A**. relative to gonadal stage in males and **B**. relative to breast developmental stage in females.

Concerns have been raised on the possibility of side effects in the future from high levels of IGF-1 because of its mitogenic effect [16,59]. Therefore, it became of interest to know the levels of IGF-1 with treatment. The levels of IGF-1 prior to treatment, during treatment for different stages of puberty, and after treatment are illustrated in Figure 4 and Table 7. Except for a few, all the values were within the range expected for the pubertal stage. Only 8.4% of the values (20 out of 237) (19 out of 198, 9.6% for the males and 2.5%, 1 out of 39, for the females) exceeded the normal range at one time during the treatment. These values are less than those reported in patients with growth hormone deficiency treated with 0.24 mg/kg/week. Seventeen percent of the values were higher than 2 SD after 2 years of treatment [60].

**Table 7:** IGF-1

**Gonadal stage**						
**Males**	**1**	**2**	**3**	**4**	**5**	**Adult**
Reference ranges* in ng/ml	**109 to 485**	**174 to 512**	**230 to 818**	**396 776**	**402-839**	**182 to 780**
**Pre treatment**						
Number	27	7				
Mean	128	250				
SD	64	81				
Minimum	27	151				
Maximum	270	178				
**Treated**						
Number	53	25	20	68	28	4
Mean	190	415	496	548	575	321
SD	127	130	162	165	184	22
Minimum	64	200	122	224	269	292
Maximum	625	637	777	961	897	338
**Post treatment**						
Number				1	2	16
Mean				374	286	321
SD					57	101
Minimum					245	178
Maximum					326	520
**Females**	**Breast stage**					
**Reference ranges in ng/ml**	**128 to 470**	**186 to 695**	**292 to 883**	**394 920**	**308-1138**	**182 to 780**
**Pre treatment**	**1**	**2**	**3**	**4**	**5**	**Adult**
Number	9	3				
Mean	167	243				
SD	80	32				
Minimum	95	214				
Maximum	331	277				
**Treated**						
Number	6	10	13	7	3	
Mean	287	327	510	662	336	
SD	95	98	192	222	63	
Minimum	146	194	207	352	284	
Maximum	397	470	959	916	406	
**Post treatment**						
Number				2	5	5
Mean				440	244	300
SD				233	52	130
Minimum				276	192	164
Maximum				605	311	511

*Reference ranges from Quest Diagnostics, Pediatric Endocrinology. Ed. Delbert A. Fisher; 2001.

### Benefit obtained by treatment

One of the limitations of our study is the lack of controls. Fortunately, there are, presently, a number of randomized and nonrandomized controlled studies that would permit assessment of the benefit, by comparisons of the adult height attained and AH gain in our treated children with those of historical untreated controls.

The adult height and adult height gain (AH minus Baseline height) are measurements that the investigators obtain and should be more accurate than comparisons based on attainments of PAH or MPH (see later). MPH was not used to calculate benefit of treatment.

The adult height gain corrects for baseline differences in the different studies in treated subjects and controls, provides information on the benefit of treatment, and permits comparison of groups not matched for baseline heights.

The comparison of adult heights provides also information on the benefit, and when the baselines heights are not different or the AHs are corrected for baseline heights differences, yields the same results as the adult height gain.

The reports on SDS permit comparisons of different populations and calculation of the benefit in centimeters based on the centimeters for SD of adults in a particular population. In this presentation we used 6.75 cm for males and 6.14 cm for females for SD of adult height, the numbers from the US National Health Statistics of 1977. The benefit in centimeters would be different in different populations depending on the centimeters for SD of adults.

**Figure 5:**
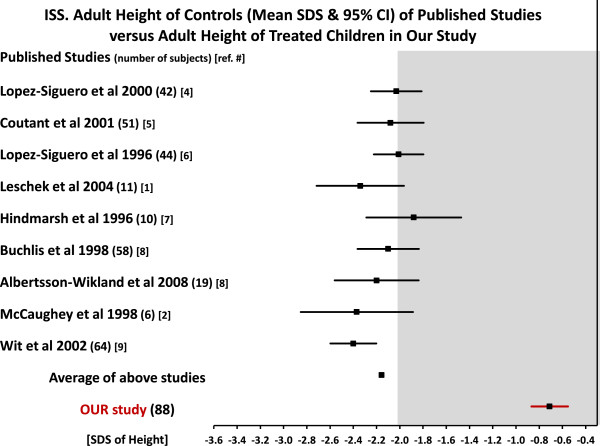
**ISS.** Adult height of controls of published studies versus adult height of treated children in our study.

1. *Adult height and adult height gain of treated children in our study versus published untreated controls.*In Figure 5 the adult height of 305 untreated controls, mean SDS and 95% CI, from 9 (3 randomized and 6 nonrandomized) controlled studies, and the adult height, mean SDS and 95% CI, of our 88 treated children are illustrated.

**Figure 6:**
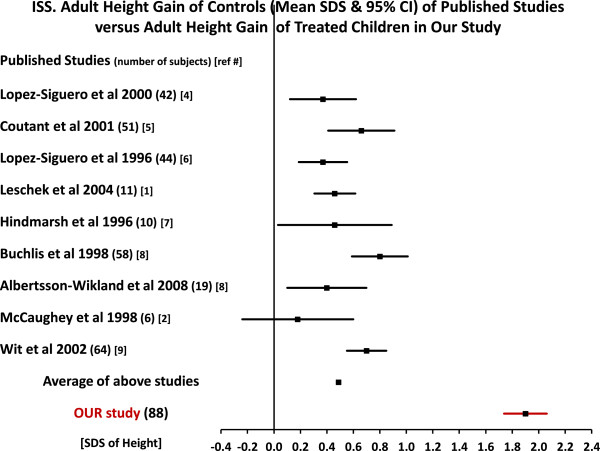
**ISS.** Adult height gain of controls of published studies versus adult height gain of treated children in our study.

In Figure 6 are similar comparisons for the adult height gain (mean SDS and 95% CI) for the controls and for our treated subjects. The numbers for the different studies for the baseline, adult height and adult height gain of controls and our treated subjects are in Table 8.A glance at the Figures 5 and 6 clearly shows that there is a significant benefit from treatment.

**Table 8:** Baseline, Adult height, & Adult height Gain of controls (Mean SDS & 95% CI) in Published studies and of Treated Children in Our Study

	**Baseline**			**Adult height**			**Adult height gain**		
**Published study [Ref. #]**	**Number**	**Mean SDS**	**(95% CI)**	**Mean SDS**	**(95% CI)**		**Mean SDS**	**(95% CI)**	
Lopez-Siguero et al. [4]	42	-2.40	-2.52 – -2.28	-2.03	-2.25 – -1.81		0.37	0.37 – 0.80	
Coutant et al. [5]	51	-2.74	-2.92 – -2.56	-2.08	-2.36 – -1.80		0.66	0.66 – 0.89	
Lopez-Siguero et al. [6]	44	-2.38	-2.50 – -2.26	-2.01	-2.22 – -1.80		0.37	0.37 – 0.60	
Leschek et al.* [1]	11	-2.80	-3.20 – -2.40	-2.34	-2.72 – -1.96		0.46	0.46 – 0.23	
Hindmarsh et al. [7]	10	-2.34	-2.78 – -1.90	-1.88	-2.29 – -1.47		0.46	0.46 – 0.60	
Buchlis et al. [8]	58	-2.90	-3.08 – -2.72	-2.10	-2.36 – -1.84		0.80	0.80 – 0.80	
Albertsson-Wikland et al.* [3]	19	-2.76	-2.95 – -2.57	-2.20	-2.56 – -1.84		0.40	0.40 – 0.62	
McCaughey et al.* [2]	6	-2.55	-2.89 – -2.21	-2.37	-2.85 – -1.89		0.18	0.18 – 0.40	
Wit et al. [9]	64	-3.10	-3.22 – -2.98	-2.40	-2.60 – -2.20		0.70	0.70 – 0.60	
**Total**	**305**								
**Mean**	**34**	**-2.66**		**-2.16**			**0.49**		
**Minimum**	**6**	**-3.00**		**-2.40**			**0.18**		
**Maximum**	**64**	**-2.34**		**-1.88**			**0.80**		
***Our study (treated)***	***88***	***-2.61***	***-2.74 – -2.48***	***-0.71***	***-0.87 – -0.55***		***1.90***	***1.74 – 2.06***	
***Difference***					***cm***			***cm***	
					***M***	***F***		***M***	***F***
***Mean***		***+0.05***		***+1.45***	***9.8***	***8.9***	***+1.41***	***9.5***	***8.6***
***Minimum***		***-0.27***		***+1.17***	***7.9***	***7.1***	***+1.10***	***7.4***	***6.7***
***Maximum***		***+0.39***		***+1.69***	***11.4***	***10.3***	***+1.72***	***11.6***	***10.5***

*Randomized controlled trials; M = male; F = Female; [#] reference number.

The benefit from treatment for the adult height for our children (-0.71 SDS) versus the AH (-2.16 SDS) of the controls of the 9 studies, Table 8, was + 1.45 SDS with a range of +1.17 SDS to +1.69 SDS (an average of 9.8 cm for males with a range of 7.9 to 11.4 cm and an average of 8.9 cm for females with a range of 7.2 to 10.3 cm).

The analysis of the adult height gain, Table 8, showed somewhat similar results. The gain of controls in the 9 studies was, on the average, +0.49 SDS (with a range of 0.18 to 0.8 SDS). In our treated children the gain was on the average +1.90 SDS for a difference with controls of +1.41 SDS, range +1.1 to +1.72 SDS (9.5 cm for males with a range of 7.4 to 11.6 cm and 8.6 cm for females, with a range of 6.7 to 10.5 cm).

The average baseline height of the 9 published studies, Table 8, was -2.66 SDS, not different than in our study -2.61 (±0.62) SDS. The baseline of 5 of the 9 studies was not different than ours p >0.1 or >0.5; in two it was higher and in two lower.

In the study of Rekers-Mombarg et al. [48] on the height outcomes of untreated children with ISS, the adult height of 48 NFSS boys of -1.74 SDS (0.91 SD) was 8.3 cm (95% CI, 7.1 to 9.5 cm), less than their target height. The adult height of our 47 treated NFSS boys of -0.54 SDS (0.64 SD) was equal to the target height, suggesting that our treated boys gained 8.3 cm. Actually, the adult height of our 47 NFSS boys of -0.54 SDS (0.64 SD) (95% CI, -0.35 to -0.73) was 1.2 SDS, 8.1 cm (6.8 to 9.3 cm) higher than the adult height of the untreated boys, indicating the benefit obtained by treatment. This benefit of 8.1 cm (6.8 to 9.3 cm) is similar to the benefit found by comparison with other studies and adds confidence in the results of our comparisons.

2. *Comparisons of our results with published Adult Heights of controls and treated children.*

Table 9 shows the benefit of different doses of growth hormone on the adult height. In published studies the benefit obtained in adult height was usually less than 4 cm over controls with doses of less than 0.3 mg/kg/week. And it was more than 5 cm (6.8, 7.4, 7.5) with doses of 0.3 or more mg/kg/week.

**Table 9:** Comparison of Our Results with Published Adult Heights of Controls & Treated Children

			**AH Controls #305**	**AH Treated #212**	**Difference**		**Benefit**	
**Published Study [Ref. #]**	**Number**	**mg/kg/wk**	**Mean SDS**	**Mean SDS**	**Mean SDS**		**Males cm**	**Females cm**
Lopez-Siguero et al. [4]	35	0.16 to 0.25	-2.03	-1.31	0.72		4.86	NA
Coutant et al. [5]	32	0.16	-2.08	-2.10	-0.02		-0.13	-0.12
Lopez-Siguero et al. [6]	20	0.16 to 0.25	-2.01	-1.46	0.55		3.71	NA
Leschek et al.* [1]	22	0.22	-2.34	-1.77	0.57		3.84	3.50
Hindmarsh et al. [7]	16	0.16 to 0.28	-1.88	-1.33	0.55		3.71	3.38
Buchlis et al. [8]	36	0.3	-2.10	-1.50	0.60	0.4 M 1.2 F	3.00	6.8
Albertsson-Wikland et al.* [3]	31	0.47	-2.20	-1.50	0.70		5.0	4.30
McCaughey et al.* [2]	8	0.35 0.4 to 0.31	-2.37	-1.14	1.23		NA	7.55
Wit et al. [9]	12	0.32 0.32 to 0.26	-2.40	-1.30	1.10		7.42	6.75
**Total number**	**212**							
**Mean**	**23.6**		**-2.16**	**-1.49**	**0.67**		**3.92**	**4.83**
**Minimum**	**8**		**-2.40**	**-2.10**	**-0.02**		**-0.13**	**-0.12**
**Maximum**	**36**		**-1.88**	**-1.14**	**1.23**		**7.42**	**7.55**
***Our study treated***	***88***	***0.32 (0.03)***	***(-2.16) (Historical)***	***-0.71***	***1.45***		***9.8***	***8.9***
***Difference***								
***Mean***				***+0.78***	***+0.78***		***5.2***	***4.8***
***Minimum***				***+0.43***	***+0.22***		***1.5***	***1.35***
***Maximum***				***+1.39***	***+1.47***		***9.9***	***9.0***

*Randomized controlled trials; M = male; F = Female.

In our study, the benefit with a dose of 0.32 (±0.03 SD) mg/kg/week was 9.8 cm for males and 8.9 cm for females, about 5 cm to 6 cm more than the 4 cm in published studies treated with less than 0.3 mg/kg/week, but, in the range, only 1.5 or 1.35 cm more than in those treated with 0.3 or more mg/kg/week.

3. *Comparisons of NFSS and FSS, treated and controls.*

There have been only 2 studies comparing NFSS and FSS, the one by Wit et al. [9] and by Albertsson-Wikland et al. [3], Table 10.

**Table 10:** Baseline, Adult height, & Adult height gain of controls (Mean SDS & 95% CI) in Published Studies of NFSS & FSS and of Treated Children in Our Study

		**Baseline**		**Adult height**			**Adult height gain**		
**Published study [Ref. #]**	**Number**	**mean SDS**	**(95% CI)**	**mean SDS**	**(95% CI)**		**mean SDS**	**(95% CI)**	
NFSS Wit [9]	45	-3.20	-3.35 – - 3.05	-2.40	-2.64 – -2.16		0.80	0.62 – 0.98	
NFSS Albertsson-Wikland [3]	36	-2.60	-2.87 – -2.33	-2.10	-2.34 – -1.86		0.50	0.23 – 0.77	
***Our study NFSS (Treated)***	***63***	***-2.62***	***-2.77 – -2.47***	***-0.56***	***-0.72 – -0.40***		***2.06***	***1.87 – 2.25***	
***Difference ― Our study to:***					***cm***			***cm***	
					***M***	***F***		***M***	***F***
*NFSS Wit*[9]		*+0.58*		*+1.84*	*12.4*	*11.3*	*+1.26*	*8.5*	*7.7*
*NFSS Albertsson-Wikland*[3]		*-0.02*		*+1.54*	*10.4*	*9.4*	*+1.56*	*10.2*	*9.5*
FSS Wit [9]	10	-2.50	-2.85 – -2.15	-2.20	-2.83 – -1.57		0.30	-0.05 – 0.65	
FSS Albertsson-Wikland [3]	10	-2.80	-3.52 – -2.08	-2.90	-3.57 – -2.23		-0.10	-0.79 – 0.59	
***Our study FSS (Treated)***	***25***	***-2.59***	***-2.86 – -2.32***	***-1.08***	***-1.41 – -0.75***		***1.51***	***1.24 – 1.78***	
***Difference ― Our study to:***					***cm***			***cm***	
					***M***	***F***		***M***	***F***
*FSS Wit*[9]		*-0.09*		*+1.12*	*7.5*	*6.9*	*+1.21*	*8.1*	*7.4*
*FSS Albertsson-Wikland*[3]		*+0.21*		*+1.82*	*12.8*	*11.2*	*+1.61*	*10.8*	*9.9*

M = male, F = female.

The benefit in adult height gain (that corrects for differences in baseline heights) of our treated children were higher than the adult height gain of the controls in the published studies by 1.26 SDS and 1.56 SDS for the NFSS (8.5 and 10.5 cm for males and 7.7 and 9.5 cm for females) and 1.21 SDS and 1.6 SDS for the FSS (8.1 to 10.8 cm for males and 7.4 to 9.8 cm for females); an equal benefit for children with NFSS and FSS.

Even though the benefit with treatment was the same for children with NFSS and FSS, our 25 children with FSS were significantly lower in adult height and adult height gain than our 63 children with NFSS, Table 11; adult height of -1.08 SDS in FSS versus -0.56 SDS in NFSS (p <0.01) and adult height gain 1.51 in FSS versus 2.06 in NFSS (p < 0.001).

**Table 11:** NFSS & FSS Adult Height Gain of Controls and Treated Subjects SDS (95% CI)

		**GH Dosage**	**Adult height gain of controls untreated**	**NFSS to FSS Difference**	**Adult height gain of treated subjects**	**NFSS to FSS Difference**
**Published study**	**No.**	**mg/kg/wk**	**Mean SDS (95% CI)**	**SDS p**	**Mean SDS (95% CI)**	**SDS p**
Wit et al. NFSS [9]	36	0.32 to 0.14	0.8 (0.62 to 0.98)	0.5 SDS p <0.01	1.50 (1.23 to 1.77)	0.7 SDS p <0.05
Wit et al. [9] FSS	5	0.32 to 0.27	0.3 (-0.05 to 0.65)		0.80 (0.06 to 1.54)	
Albertsson-Wikland et al. NFSS [3]	48	0.47	0.5 (0.23 to 0.77)	0.6 SDS p <0.05	1.40 (1.17 to 1.63)	0.5 SDS p < 0.05
Albertsson-Wikland et al. [3] FSS	14	0.47	-0.1 (-0.79 to 0.59)		0.90 (0.44 to 1.36)	
			***Adult height***		***Adult height gain***	
Our Study NFSS (treated)	63	0.32 (0.03)	-0.56 (-0.72 to -0.40)	0.52 SDS p < 0.01	2.06 (1.87 to 2.25)	0.55 SDS p <0.001
Our Study FSS (treated)	25	0.32 (0.03)	-1.08 (-1.41 to -0.75)		1.51 (1.24 to 1.78)	

Of interest is that the results in the only two studies that have been published are similar, Table 11. The adult height gain of the untreated controls and of the treated subjects were significantly less in the children with FSS than in NFSS (p <0.05). In other words, the adult height gain of treated children with NFSS was higher than in FSS, but also higher was the adult height gain of the NFSS than the FSS controls, so the benefit was the same. This was also the conclusion of Dahlgren J [12] in his analysis of these 2 studies.

This observation seems to be consistent in the three different studies and has implications for interpretation of results with treatment, in studies where the number of children with FSS in the controls and treated subjects is not known, and in the selection of controls for non-randomized or randomized studies.

4. *Other Comparisons.*

The reported impression, by review of published studies, is that the benefit of treatment of children with ISS is less than in other conditions for which GH has been licensed [20,21,61].

Our results compare well with those obtained in GH treated children in other conditions for which GH has been approved, growth hormone deficiency (GHD), small for gestational age (SGA), or Turner syndrome.

The adult height of our 88 children with ISS of -0.71 SDS (0.74 SD) is equal to the adult height of -0.7 SDS (1.2 SD) achieved in 121 children (males and females) with GHD treated with 0.3 mg/kg/week, 3 or 6 times a week, reported by Blethem et al. [62].

In a review of four randomized controlled trials (RCTs) comprising 391 children with SGA treated with growth hormone (range of 0.23 to 0.47 mg/kg/week) [63], the adult height exceeded controls by 0.9 SDS. The adult height gain was 1.25 SDS (1.5 SDS in treated minus 0.25 SDS in untreated subjects). There was no difference between the 2 dose regimens. The adult height gain in our study exceeded controls by 1.41 SDS (1.90 SDS minus 0.49 SDS in controls), Table 8.

Our results of an increase of 7.1 to 10.3 cm of adult height in treated females over controls (Table 8) compares favorably with those obtained in Turner syndrome. In 61 patients with Turner syndrome [64], treated with 0.3 to 0.375 mg/kg/week of growth hormone, the final height was approximately seven cm higher (95% CI, of 6 to 8 cm) than in 43 untreated control patients. Despite this increase, the adult height of treated patient with Turner syndrome was still outside the normal range.

## Discussion

Our experience with the growth hormone treatment of children with ISS was a positive one, with the normalization of the height and growth during childhood and adolescence after 2 or 3 years of treatment and the attainment of a normal adult height of -0.71 SDS (0.74 SD) (95% CI -0.87 to -0.55), the main two aims of treatment; an adult height from the first percentile to the 80^th^ percentile for males and for females.

The treatment was safe. There were no significant adverse effects. The IGF-1 values were essentially within the levels expected for the stages of puberty.

All the parents of the children and the children in our study who attained adult heights were pleased with the results, happy for their children to be normal and grateful for the treatment. The most consistent perception of the parents of the benefit was the improvement of self-esteem in their children, and of the children to be happy to be normal and not different. Other benefits perceived were cessation of teasing, bullying and psychological stress.

The reason for the children lost to follow-up, all growing well and benefiting from treatment, is not known. One can speculate that some were content to be within normal range in height, some may have had difficulty with copays, may have moved, did not care to have more injections, even though all reported that the injections were not painful, and so on.

The benefit obtained in our study seems to be better than that reported in a number of previous studies and there must be some reason. Care was taken in the selection of children to be certain they met qualifications, in the measurements, in the analysis of data and compilation of results.

By our observations, there may be two possible reasons, among others; namely the dose of growth hormone used (dose dependent effect), and the effect of including an unknown number of children with FSS in the group in previous studies.

### Benefit of GH treatment – Effect of GH dose

Our observations in 88 children clearly show a significant benefit of GH treatment (0.32 ± 0.03 mg/kg/week) when compared to 305 untreated controls in 9 different studies, a benefit based on adult height gain (that corrects for baseline differences) of 9.5 cm (7.4 to 11.6) for males and 8.6 cm (6.7 to 10.5) for females (Table 8).

The benefit in the adult height of our treated subjects over the adult height of published historical controls was 9.8 cm for males and 8.9 cm for females (Table 9). This benefit is higher by 5 or 6 cm than the benefit obtained in subjects treated with 0.16 or 0.16 to 0.28 mg/kg/week of GH, usually of less than 4 cm. The benefit was only 1.35 or 1.5 cm higher than that obtained in other studies using 0.3 mg/kg or more per week.

This dose dependent effect has been reported previously in studies using 0.3 mg/kg or more of GH a week [65]. A dose effect for children with ISS was shown by Wit et al. [66] with a mean adult height gain of 1.85 SDS (similar to ours) and a benefit of 7.2 cm with a dose of 0.37 mg/kg/week. Also in a randomized controlled study by McCaughey et al. [2], 8 girls (6 of them with FSS) treated with GH 0.35 mg/kg/week (range 0.4 to 0.31), were 7.5 cm taller than the controls; and by others: Buchlis et al. [8] 6.8 cm for females, Albertsson-Wikland et al. [3] 8 cm based on adult height gain, Hintz R et al. [12] 9.2 cm higher for boys and 5.7 cm for girls, and Kemp et al. [67] (Genentech registry) 1.7 to 2.0 SDS in AH gain in children treated for 7 years, results similar to ours.

In view of the aforementioned, it is possible that the stated modest benefit of 4 cm with the treatment of ISS is related to the low dose of GH used and to averaging the results without taking in consideration the difference in the benefit from different doses.

In the meta-analysis conducted by Finkelstein et al. [10], there were only four controlled studies reporting adult height, -1.51 SDS. The reported benefit of treatment over controls, on the average was 5.7 cm, range 2.3 to 8.7 cm, a wide range. Two of the studies used dosages of GH of 0.25 and 0.26 mg/kg/week and the other two doses of 0.3 and 0.4 mg/kg/week, which may account for the 8.7 cm benefit.

In the review of Guyda H et al. [68], comprising 413 treated children in 11 studies the average adult height was -1.7 SDS (range -1.1 to -2.4 SDS). Seven of the studies used doses of GH ranging from 0.21 to 0.26 mg/kg/week for an average of 0.24 mg/kg/week. The difference of the adult height range from -1.1 to -2.4 SDS is 1.3 SDS, a large difference, that could be owing to a difference in the GH dose used. In three of the studies using 0.3 mg/kg/week or more the adult height was -1.1, -1.3, and -1.4 SDS. So the difference of adult height from -1.4 to -2.4 SDS was owing mainly to studies using the lower dose of GH.

As a consequence of the averaging, the improved benefit obtained, in SDS or cm, with the higher doses of GH is not reflected in the reports, as is illustrated in Table 9, averaging results of published studies with no benefit (-0.1 cm) with others with a benefit of 7.5 cm.

### The effect of the inclusion of children with FSS

By our results, the adult height and adult height gain were significantly higher by 0.6 SDS (4 cm) in the 47 treated males with NFSS than in the 21 with FSS (Table 4), and in our 63 (males and females) with NFSS (0.55 SDS) higher than in the 25 (males and females) with FSS (Tables 4 and 11).

Similar results were obtained in the only 2 studies reporting the results of NFSS and FSS. The adult height gain of the untreated controls and of treated subjects was significantly higher in the children with NFSS than in FSS (Table 11).

This seems to be a consistent finding in different studies and has implications for the interpretation of the results observed in published studies. The inclusion of a high number of children with FSS in the treated cohort will yield lower results. And also, it has implications for the selection of controls in randomized or non-randomized controlled studies, since the number of children with FSS in the treated or control groups may affect the results.

It is difficult to assess how this has affected the results of previous studies, since most or practically all of the published studies included all the children as ISS and did not mention whether they were NFSS or FSS.

It would seem advisable to identify the two groups NFSS and FSS in future trials.

### Assessment of Benefit

The assessment of the benefit from treatment with GH is sometimes difficult, even in controlled studies, because the proportion of children with FSS and NFSS, normal and delayed puberty, and at times IUGR, may not be the same in the treated and control groups.

As previously mentioned, to assess the benefit of treatment, comparison of the AH gain (adult height minus the baseline height) and the AH attained of the treated and control groups would seem to be the best method, because they are based on measurements by the investigator.

Also useful would be the comparison between the groups on the attainment of target height. Target heights, however, are often based on self-reported parental heights. In addition, children with FSS may attain heights near or equal to the MPH without treatment, so the inclusion of a different number of children with FSS in the group may influence the attainment of MPH and render the interpretation as benefit of treatment inaccurate. Furthermore, there are a number of reported methods to calculate MPH [47], which may give different results for the benefit when comparing AHs and MPHs. It would also be difficult to compare the benefit of different studies using different methods for calculating MPH.

Calculation of the benefit to children with ISS, treated with GH, based on the difference of AH attained and PAH at the baseline does not appear accurate. The imprecision and inaccuracy of PAH for both sexes have been indicated in a number of reports and only 33% of the variability in achieved AH is predictable. Commonly used methods tend to over-predict AH, especially in young males and in children with delayed bone age, and under-predict if the bone age is not delayed [69]. Also, the individual variability of the tempo of progression of the bone age, either faster or slower than usually expected, as illustrated in Figure 1, affects the accuracy of the PAH. In a randomized controlled trial, girls with ISS treated with GH were 3.5 cm taller than the originally PAH, but 7.5 cm taller than controls when calculated on the basis of AH and AH gain in SDS, a meaningful difference [2].

### Other considerations

Recent critical reviews [20,21,61] concluded that to date no study has fulfilled the criteria for high quality and strong recommendations, in part owing to the small number of children in the studies, and felt that additional high quality trials up to the achievement of adult height would be necessary to determine the efficacy, ideal dosage, long term safety of growth hormone therapy and to address health related quality of life and cost issues.

There is agreement with their recommendations. Even though rhGH has proven to be a remarkably safe medication for 27 years at the doses recommended, long surveillance studies have been suggested by many, because of the mitogenic effect of IGF-1.

Regarding the dose, there has been many and probably enough trials with a dose of less than 0.3 mg/kg/week of GH, to know that it is not an adequate dose to induce a meaningful or satisfactory gain. Doses higher than 0.3, whether it be 0.32, 0.35, 0.37 mg/kg/week, or starting with 0.32 with adjustments may give a satisfactory gain, but the studies have been few; so additional studies would be helpful. It may be preferable to calculate the dose on mg/kg/week; calculations based on m^2^ of surface area, would provide higher doses per kg at 6 years of age than later because of the nonlinear relationship of m^2^ of surface area and weight.

Additional genetic studies may give useful information to explain the variability of response.

In the past 27 years, since rhGH became available in 1985, there have been 3 or 4 randomized studies, up to the achievement of adult height, and, apparently, they did not provide the answers. It may take 8 or more years to get results from more randomized trials. It is hoped that we do not wait for the answer; many children could benefit while we wait.

One of the main problems that has been often addressed in the past is the significant cost, which limits the availability to children who may need it, raises questions about the use of health resources, and a number of ethical considerations [25]. One may question whether studies would seem applicable to solve the problem of cost; participation from the pharmaceutical industry would be required. The high cost of biopharmaceuticals is a problem concerning public health services around the world, is in the public domain^a^, and to reduce cost and increase affordable access to treatment may not be as simple as one may think [70-72].

The wholesale acquisition cost (the list price paid by the distributing pharmacies from the supplier), generally, the price put by the manufacturer of the GH was $50,000 per gram in 2007 [73] and apparently $76,000 in 2010 [74]. The cost to the patients (or insurances) for the purchase of GH from the distributing pharmacies, presently, may be as much as $88,000 to $100,000 per gram. This is based on the pharmacy bills given to the family for the purchase of GH^b^.

The estimated cost of GH therapy compared with no therapy, in 2006, was $52,634 per inch (2.54 cm) or $99,959 per child reflecting an incremental growth of 1.9 in (4.8 cm) (Lee JM et al.) [22] and by others, in 2011, at $113,000 per inch or more (Durand-Zaleski I et al.) [23], depending on unit cost and height gain. This cost is applicable to any child treated with GH, whether it be growth hormone deficiency, Turner syndrome, intrauterine growth retardation or ISS.

If the price was reasonable, many of the objections to treatment, concerns for use of heath resources, and ethical considerations would subside. Cost influences pediatric endocrinologists in their decision to treat [75], and third party payers (private insurances or health agencies, state or national) in their decision to support treatment [74]. Also, if the price was reasonable, it, probably, would be the right thing to do to help the children to attain an adult height within the range judged to be normal by National Health Standards and by society. It would not harm anybody.

## Conclusion

Children with ISS do not attain a normal adult height: adult heights of -2.4 SDS (0.8 SD) to -1.88 SDS (0.57 SD). Growth hormone treatment significantly increases the adult height, but the benefit obtained with doses of less than 0.3 mg/kg/week is modest, usually less than 4 cm.

The benefit obtained seems dose dependent and a benefit of 7, 7.5, and 8 cm have been reported with higher doses of 0.32 to 0.4 mg/kg/week, but the studies are few.

We conducted a retrospective analysis of our experience with children with ISS treated with rhGH, 0.32 ± 0.03 mg/kg/week. The treatment was quite helpful with normalization of the height and growth during childhood and attainment of normal adult heights. Eighty eight (68 males, 20 females) attained an adult height of -0.71 SDS (0.74 SD) (95%CI -0.87 to -0.55), a benefit over 305 untreated historical controls in 9 different studies of 9.5 cm (7.4 to 11.6 cm) for males and 8.6 cm (6.7 to 10.5 cm) for females, with heights from the 1^st^ to the 80^th^ percentile for males and females.

In the analysis of the subgroups the adult height and adult height gain of children with NFSS were significantly higher than of FSS. Similar results were obtained in the only 2 studies previously reported.

No difference was found in the cohorts with normal or delayed puberty in any of the subgroups, except between the NFSS and FSS subgroups. This has implications for the interpretation of the benefit of treatment in studies where the number of children with FSS in the controls or treated subjects is not known. It would seem advisable to identify the two groups, NFSS and FSS, in future trials.

The treatment was safe. There were no significant adverse events. The IGF-1 values were essentially within the levels expected for the stages of puberty.

There have been, probably, enough trials with a dose of less than 0.3 mg/kg/week to know that it is not an adequate dose to induce a meaningful or satisfactory gain. Studies with doses from 0.3 to 0.375 mg/kg/week are few, so additional trials would be helpful.

Additional high quality studies have been suggested to determine the efficacy, ideal dosage, health related quality of life, long term safety of GH therapy, and cost. There is agreement among investigators for these recommendations. In the past 27 years, since rhGH became available in 1985, there have been 3 or 4 randomized studies to adult height, but, apparently, did not provide the answers. It may take 8 or more years to get results from more randomized trials. It is the hope that we do not wait for the answer; many children could benefit while we wait.

## Endnotes

^a^The high price of pharmaceuticals is in the public domain. See The Economist (Economist.com), January 4^th^ – 10^th^, 2014 – Pharmaceutical pricing, arguments over the cost of drugs are growing – page 10 and 45–46 and January 25^th^ – 31^st^ – Protect patients not patents – page 14.

^b^The pharmacy bill for the purchase of 5 pens, each containing 20 mg of rhGH, (100 mg) was $8,800 every month, $88,000 per gram. Another pharmacy bill for the purchase of a pen containing 15 mg/of rhGH was $1,500, $100,000 per gram.

## Abbreviations

AH: Adult height; AH gain: Adult height gain; CBC: Complete blood count; CDGP: Constitutional delay of growth and puberty; CI: Confidence interval; DP: Delayed puberty; F: Female; FDA: Food and Drug Administration; FSS: Familial short stature; GH: Growth hormone; GHD: Growth hormone deficiency; GHR: Growth hormone receptor; IGF-1: Insulin like growth factor 1; IGFBP-3: Insulin like growth factor binding protein 3; ISS: Idiopathic short stature; kDa: Kilodalton; M: Male; MPH: Mid-parental height; NFSS: Non-familial short stature; NP: Normal puberty; PAH: Predicted adult height; rbGH: Recombinant bovine growth hormone; rbST: Recombinant bovine somatotropin; RCT: Randomized control trials; rhGH: Recombinant human growth hormone; RIA: Radioimmunometric assay; SD: Standard deviation; SDS: Standard deviation score; SGA: Small for gestational age; T_4_: Thyroxine; TSH: Thyroid stimulating hormone.

## Competing interests

The authors declare that they have no competing interests.

## Authors’ contributions

JFS contributed to conception and design, treatment of subjects, acquisition, analysis and interpretation of data, drafting and final approval of the manuscript and agrees to be accountable for all aspects of the work. NJT contributed to testing, acquisition, analysis, and interpretation of data, drafting of the manuscript and final approval of the version to be published.

## Authors’ information

JFS is Professor of Pediatrics at The Ohio State University, College of Medicine and member of the Section of Pediatric Endocrinology, Metabolism and Diabetes at Nationwide Children’s Hospital, Columbus, Ohio. NJT is a research associate of the Section of Endocrinology, Metabolism and Diabetes at Nationwide Children’s Hospital, Columbus, Ohio.

## Supplementary Material

Additional file 1: Table S1 The adult heights reported were adult or near adult heights. There was a potential growth remaining of children whose heights were obtained before closure of the epiphyses (near adult height) and before growth ended. Based on the last bone age available on the record, the predicted adult height was calculated (i.e. male with bone age of 16 years – predicted adult height (PAH), 3.0 cm more) and taking into account, if exceeded the adult height available in the record. The effect on the average adult height of the group is shown. This potential growth remaining was not included in the figures reported for adult heights, but could be taken into consideration to determine final adult height and the benefit of growth hormone treatment. Click here for file

Additional file 2: Figure S1 Correlation of GH measurement in 891 serum samples by Kallestad (polyclonal antibody - RIA) and Hybritech (monoclonal antibody immunoradiometric assay) in our laboratory at Children’s Hospital (now Nationwide Children’s Hospital (NCH)). Genentech was kind enough to provide us with the correlation they obtained in their laboratory of GH samples assayed by both methods. The correlations indicate that the 22 kDa GH measurement with a monoclonal antibody yields values that are 64 to 68% of those that are assayed by the polyclonal antibody method. Click here for file

Additional file 3: Figure S2 A, B, C, D The individual values for the heights at the beginning of treatment, adult heights, midparental heights, age at the beginning of treatment and when the adult heights were obtained, and means ± standard deviations for different subgroups are shown: A. All ISS males, B. NFSS males, C. FSS males, D. All ISS females. Click here for file

Additional file 4: Figure S3 A, B In the gray is the growth chart of the US National Health Statistics of 1977. The dark lines are the mean ± 2 of the Rekers-Mombarg et al study reported in 1996 [48] of untreated children with ISS. The results of the individual heights prior to treatment and AHs are plotted at the respective ages after treatment and the MPHs are plotted to the right: A. ISS males, B. ISS females. Click here for file

Additional file 5: Figure S4 Correlations of AH gain relative to age at the start of GH treatment and correlations of AH gain relative to duration of GH treatment for 47 NFSS and 21 FSS males. Click here for file
