# Supplementary material for: Growth hormone significantly increases the adult height of children with idiopathic short stature: comparison of subgroups and benefit
Source: Int J Pediatr Endocrinol. 2014 Jul 16;2014(1):15. doi: 10.1186/1687-9856-2014-15 (PMC4114101; doi:10.1186/1687-9856-2014-15)
Supplement: Additional file 1: Table S1 — The adult heights reported were adult or near adult heights. There was a potential growth remaining of children whose heights were obtained before closure of the epiphyses (near adult height) and before growth ended. Based on the last bone age available on the record, the predicted adult height was calculated (i.e. male with bone age of 16 years – predicted adult height (PAH), 3.0 cm more) and taking into account, if exceeded the adult height available in the record. The effect on the average adult height of the group is shown. This potential growth remaining was not included in the figures reported for adult heights, but could be taken into consideration to determine final adult height and the benefit of growth hormone treatment. [file 1687-9856-2014-15-S1.docx]

**Table S1-**

| **Determination of Adult Heights** | | | | |
| --- | --- | --- | --- | --- |
| **The Effect on the Average Adult Height of the Group** | | | | |
| **Males** | | | | |
| For all 68 males | | 1.90 cm more | | = 0.28 SDS |
| For NFSS (20) with normal puberty | | 1.49 cm more | | = 0.22 SDS |
| For NFSS (27) with delayed puberty | | 2.02 cm more | | = 0.30 SDS |
| For FSS (21) with normal & delayed puberty | | 2.11 cm more | | = 0.31 SDS |
| **Females** | | | | |
| For all 20 females | | 0.73 cm more | | = 0.12 SDS |
| **Final Adult Height (Near Adult Height plus Remaining Growth)** | | | | |
| **Group** | **AH or**  **Near AH** | **Remaining Growth**  **+ cm = SDS** | **AH SDS** | |
| **Males** |  |  |  | |
| All 68 males | -0.72 | + 1.90 = 0.28 | -0.44 | |
| NFSS (20) NP | -0.60 | + 1.49 = 0.22 | -0.38 | |
| NFSS (27) DP | -0.49 | + 2.02 = 0.30 | -0.19 | |
| FSS (21) NP & DP | -1.14 | + 2.11 = 0.31 | -0.83 | |
| **Females** |  |  |  | |
| All (20) NP & DP | -0.65 | + 0.73 = 0.12 | -0.53 | |
|  |  |  |  | |
| **All Males & Females (88)** | -0.71 | +1.46 = 0.22 | -0.49 | |

**Legend for Table**

The adult heights reported were adult or near adult heights. There was a potential growth remaining of children whose heights were obtained before closure of the epiphyses (near adult height) and before growth ended. Based on the last bone age available on the record, the predicted adult height was calculated (i.e. male with bone age of 16 years – predicted adult height (PAH), 3.0 cm more) and taking into account, if exceeded the adult height available in the record. The effect on the average adult height of the group is shown. This potential growth remaining was not included in the figures reported for adult heights, but could be taken into consideration to determine final adult height and the benefit of growth hormone treatment.
