# Supplementary figures and images for: Growth hormone significantly increases the adult height of children with idiopathic short stature: comparison of subgroups and benefit
Source: Int J Pediatr Endocrinol. 2014 Jul 16;2014(1):15. doi: 10.1186/1687-9856-2014-15 (PMC4114101; doi:10.1186/1687-9856-2014-15)

# Kallestad vs Hybritech GH Assay (Polyclonal to monoclonal assay)

## Correlations

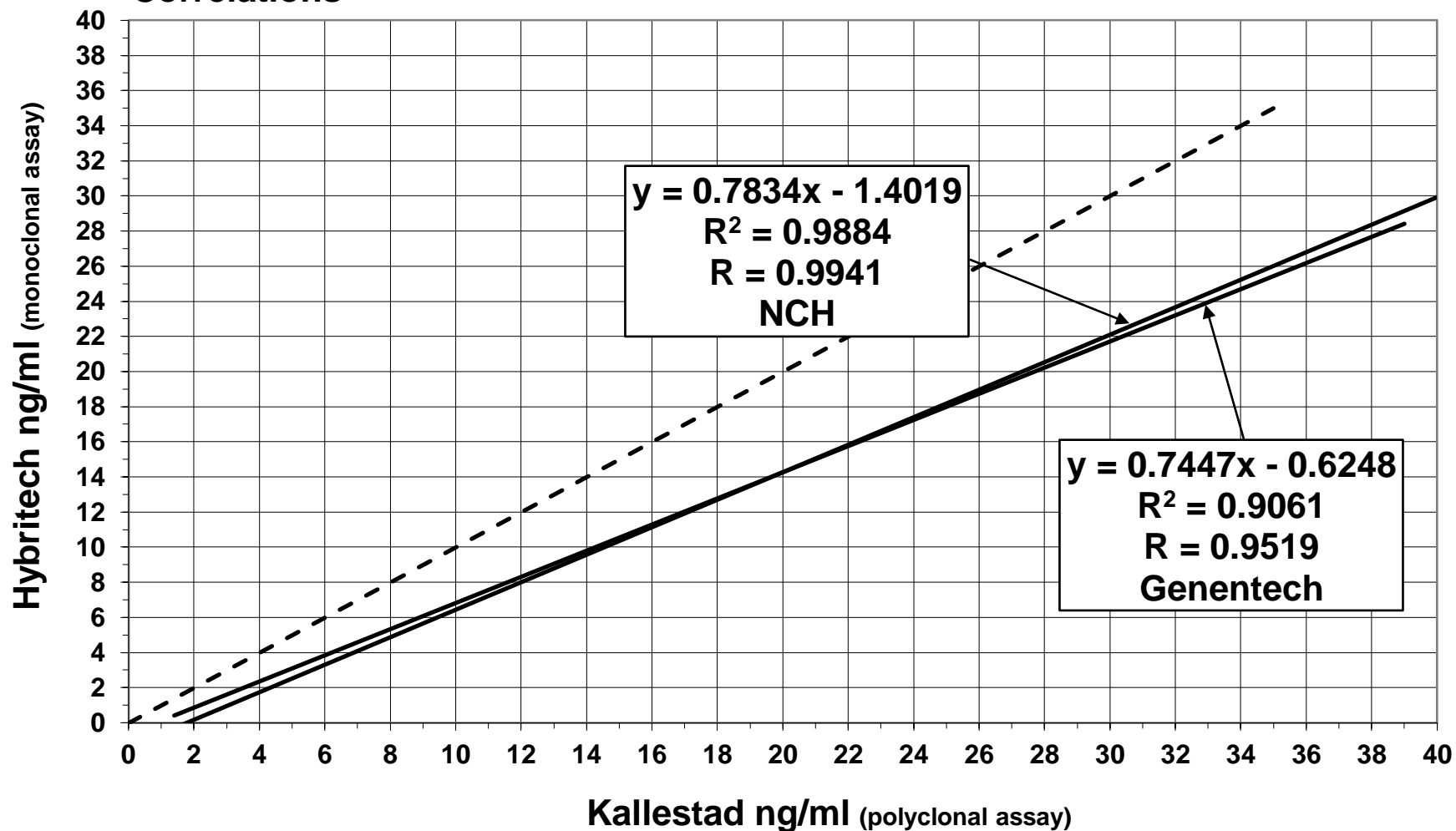

Supplement: Additional file 2: Figure S1 — Correlation of GH measurement in 891 serum samples by Kallestad (polyclonal antibody - RIA) and Hybritech (monoclonal antibody immunoradiometric assay) in our laboratory at Children’s Hospital (now Nationwide Children’s Hospital (NCH)). Genentech was kind enough to provide us with the correlation they obtained in their laboratory of GH samples assayed by both methods. The correlations indicate that the 22 kDa GH measurement with a monoclonal antibody yields values that are 64 to 68% of those that are assayed by the polyclonal antibody method. [file 1687-9856-2014-15-S2.pdf]

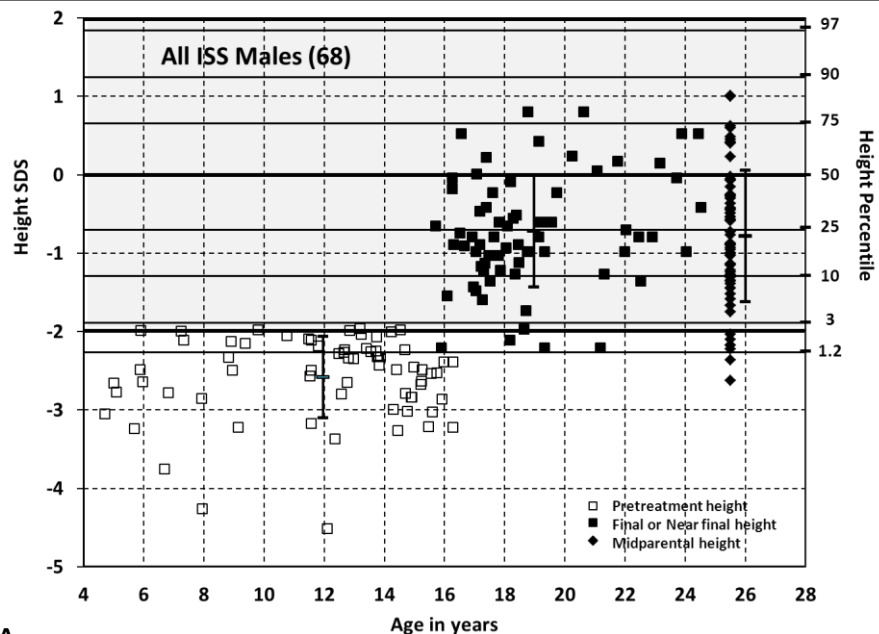

A

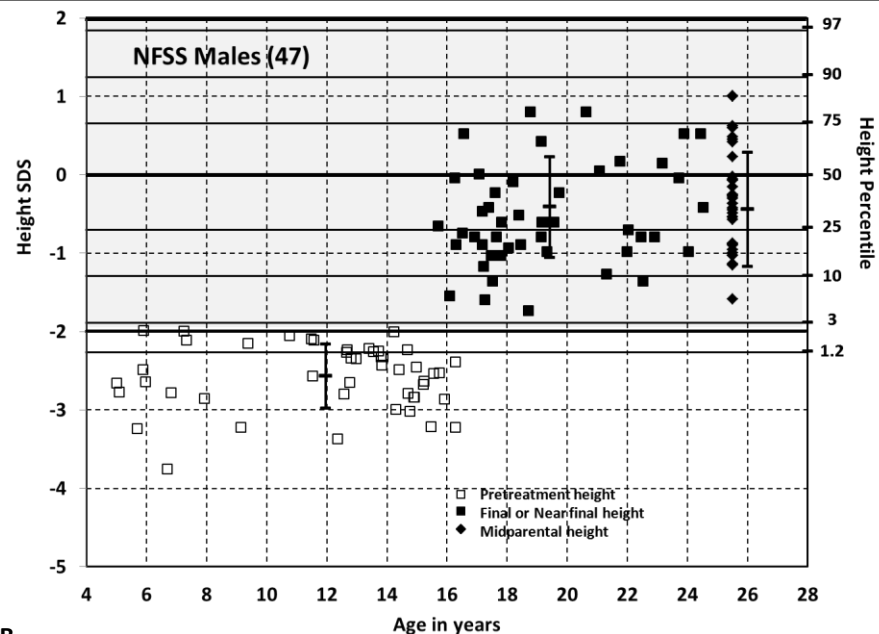

B

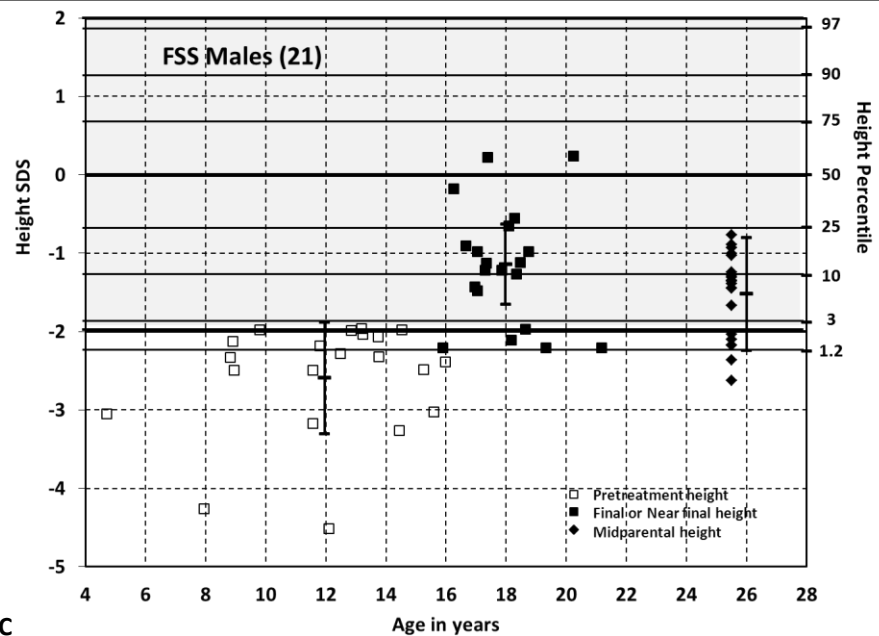

C

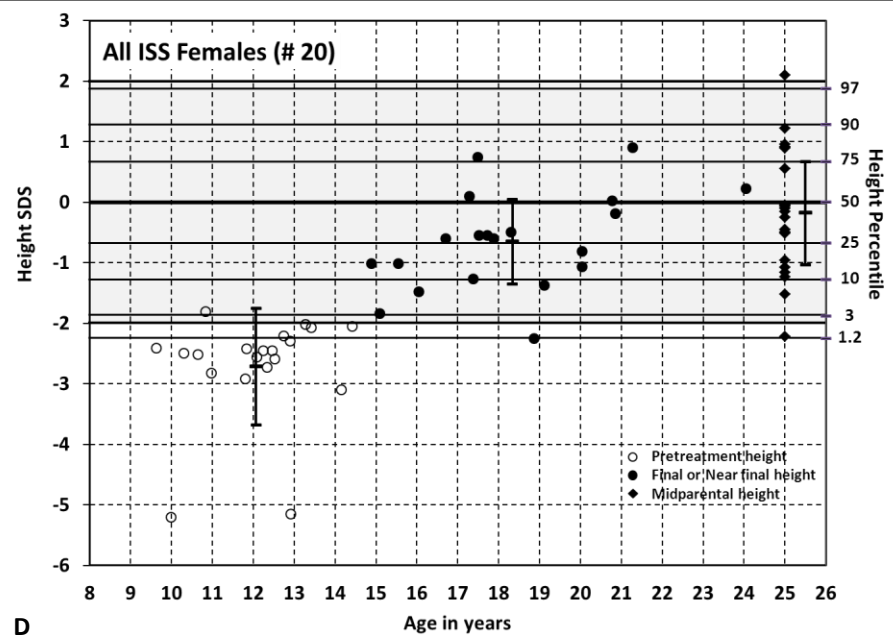

D

Supplement: Additional file 3: Figure S2 A, B, C, D — The individual values for the heights at the beginning of treatment, adult heights, midparental heights, age at the beginning of treatment and when the adult heights were obtained, and means ± standard deviations for different subgroups are shown: A. All ISS males, B. NFSS males, C. FSS males, D. All ISS females. [file 1687-9856-2014-15-S3.pdf]

### Male ISS (# 68)

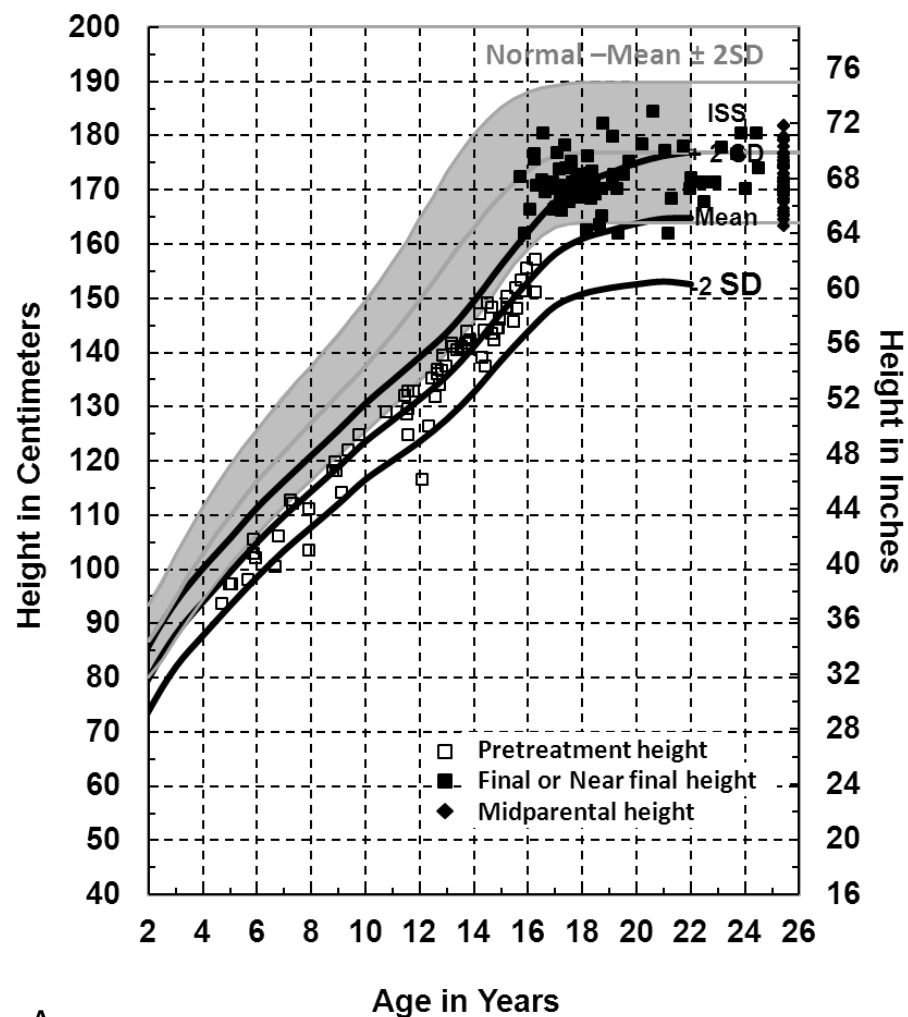

### Females ISS (# 20)

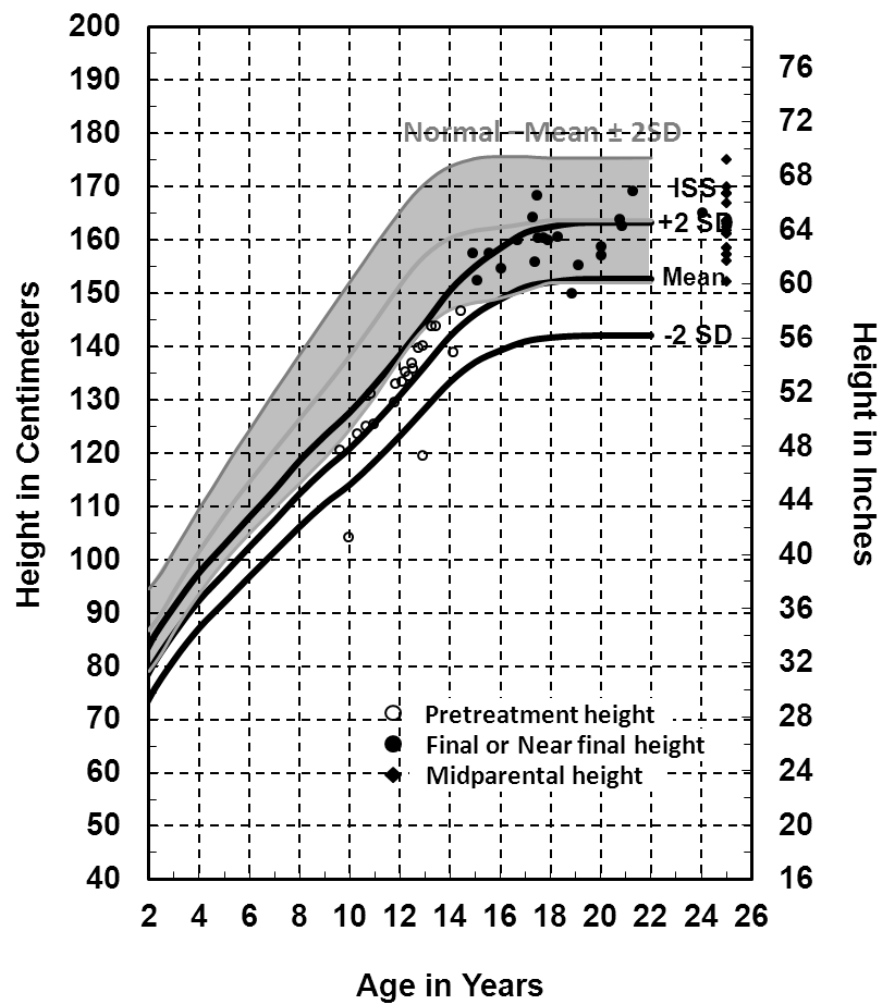

Supplement: Additional file 4: Figure S3 A, B — In the gray is the growth chart of the US National Health Statistics of 1977. The dark lines are the mean ± 2 of the Rekers-Mombarg et al study reported in 1996 [48] of untreated children with ISS. The results of the individual heights prior to treatment and AHs are plotted at the respective ages after treatment and the MPHs are plotted to the right: A. ISS males, B. ISS females. [file 1687-9856-2014-15-S4.pdf]
