# Supplementary material for: Growth hormone significantly increases the adult height of children with idiopathic short stature: comparison of subgroups and benefit
Source: Int J Pediatr Endocrinol. 2014 Jul 16;2014(1):15. doi: 10.1186/1687-9856-2014-15 (PMC4114101; doi:10.1186/1687-9856-2014-15)
Supplement: Additional file 5: Figure S4 — Correlations of AH gain relative to age at the start of GH treatment and correlations of AH gain relative to duration of GH treatment for 47 NFSS and 21 FSS males. [file 1687-9856-2014-15-S5.pdf]

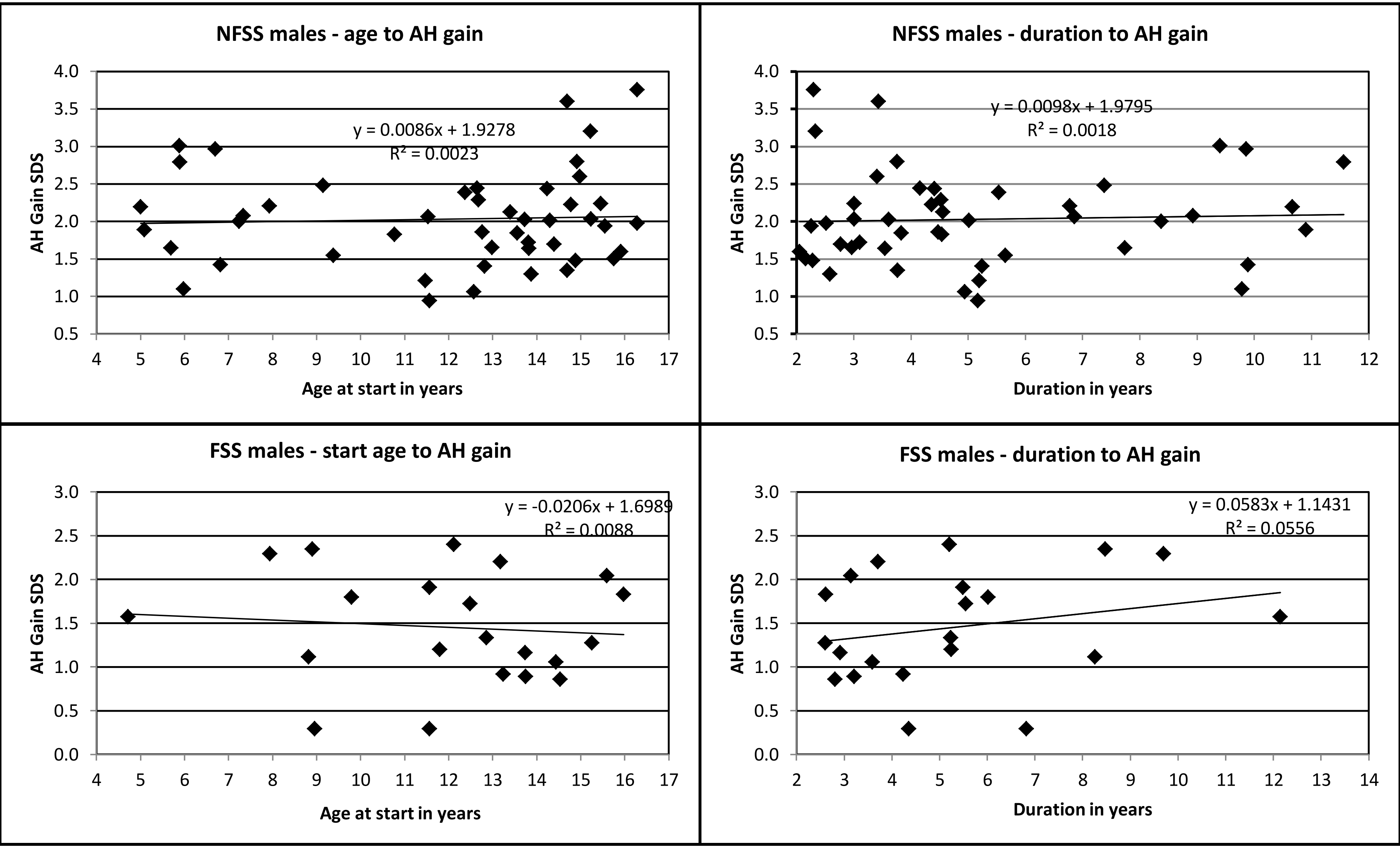

NFSS males - age to AH gain
y = 0.0086x + 1.9278
R² = 0.0023
AH Gain SDS
Age at start in years
NFSS males - duration to AH gain
y = 0.0098x + 1.9795
R² = 0.0018
AH Gain SDS
Duration in years
FSS males - start age to AH gain
y = -0.0206x + 1.6989
R² = 0.0088
AH Gain SDS
Age at start in years
FSS males - duration to AH gain
y = 0.0583x + 1.1431
R² = 0.0556
AH Gain SDS
Duration in years
